# Wear Particles Derived from Metal Hip Implants Induce the Generation of Multinucleated Giant Cells in a 3-Dimensional Peripheral Tissue-Equivalent Model

**DOI:** 10.1371/journal.pone.0124389

**Published:** 2015-04-20

**Authors:** Debargh K. Dutta, Pushya A. Potnis, Kelly Rhodes, Steven C. Wood

**Affiliations:** 1 Department of Biology, Chemistry and Materials Science, Office of Science and Engineering Laboratories, CDRH, FDA, Silver Spring, Maryland, United States of America; 2 Department of Medicine, Uniformed Services University of the Health Sciences, Bethesda, Maryland, United States of America; 3 University of Maryland, College Park, Maryland, United States of America; Rutgers—New Jersey Medical School, UNITED STATES

## Abstract

Multinucleate giant cells (MGCs) are formed by the fusion of 5 to 15 monocytes or macrophages. MGCs can be generated by hip implants at the site where the metal surface of the device is in close contact with tissue. MGCs play a critical role in the inflammatory processes associated with adverse events such as aseptic loosening of the prosthetic joints and bone degeneration process called osteolysis. Upon interaction with metal wear particles, endothelial cells upregulate pro-inflammatory cytokines and other factors that enhance a localized immune response. However, the role of endothelial cells in the generation of MGCs has not been completely investigated. We developed a three-dimensional peripheral tissue-equivalent model (PTE) consisting of collagen gel, supporting a monolayer of endothelial cells and human peripheral blood mononuclear cells (PBMCs) on top, which mimics peripheral tissue under normal physiological conditions. The cultures were incubated for 14 days with Cobalt chromium alloy (CoCr ASTM F75, 1–5 micron) wear particles. PBMC were allowed to transit the endothelium and harvested cells were analyzed for MGC generation via flow cytometry. An increase in forward scatter (cell size) and in the propidium iodide (PI) uptake (DNA intercalating dye) was used to identify MGCs. Our results show that endothelial cells induce the generation of MGCs to a level 4 fold higher in 3-dimentional PTE system as compared to traditional 2-dimensional culture plates. Further characterization of MGCs showed upregulated expression of tartrate resistant alkaline phosphatase (TRAP) and dendritic cell specific transmembrane protein, (DC-STAMP), which are markers of bone degrading cells called osteoclasts. In sum, we have established a robust and relevant model to examine MGC and osteoclast formation in a tissue like environment using flow cytometry and RT-PCR. With endothelial cells help, we observed a consistent generation of metal wear particle- induced MGCs, which heralds metal on metal hip failures.

## Introduction

Metal-on-Metal (MoM) hip implants comprised of cobalt and chromium alloy were introduced in1953 with the intent to provide greater strength to the lining of large sized head and cup of the prosthesis to replace traditional polyethylene implants[1]. In addition, a MoM hip implant was designed to reduce the release of wear metal particles from the inner linings and to minimize metal toxicity [2,3]. However, recent data suggests that MoM prostheses are failing at increased rates and are associated with an amplified rate of revised arthroplasty [4]. It is projected that the demand for hip arthroplasty will increase by 174%, reaching up to a total of 572,000 patients by 2030 [5]. The wearing of cobalt and chromium implants causes the release of metal ions, metal particles and debris into the surrounding tissue, wherein metal ions breach the tissues and enter the bloodstream. Patients present with pain, limited motion of the prosthetic limb and magnetic resonance imaging confirms osteolysis [6]. Histological examination of soft tissues surrounding MoM prostheses revealed cystic masses, denoted by granulomatous lesions comprised of histiocytes, multinucleated giant cells (MGCs) and few plasma cells [7]. Several patients with pain also had evidence of soft tissue lesions (pseudo-tumor formation) around MoM hip implants[6], and a hypersensitive reaction to metal was the probable cause. Indeed, metal particles were found to be accumulated in the surrounding tissues and such patients showed disrupted synovial lining integrity, inflammatory cell infiltrates and necrosis [8]. Aseptic loosening of the prosthetic joints is typically due to cellular reactions (such as inflammatory and immunological responses) to the presence of metal particles and debris in the implant bed [9]. Thus, it appears that biological mechanisms rather than mechanical problems, such as fracture or geometry of the head, neck and cup of the implant, are responsible for MoM implant failures.

Under normal physiological conditions, bone structure and growth (homeostasis) are regulated by the balanced activity of osteoblasts (bone generating cells) and osteoclasts (bone degrading cells). Monocytes and macrophages, the primary sentinels of innate immunity, exhibit phagocytic activity and elicit danger signals (cytokines and chemokines) in response to foreign bodies. Like osteoclasts, macrophages are capable of bone degradation. Histological evidence from arthroplasty patients showed that osteoclasts occupied 7.67± 1.82% of the bone surface, while macrophages covered 19.33± 5.26% of the bone surface [10]. Patients with osteolysis showed macrophages occupying 33.37± 8.59% of the bone surface as compared to 5.29± 1.34% of those without osteolysis [11].

During aseptic loosening, endothelial cells in tissues at the implant interface upregulate selectins and adhesion molecules, such as P-selectin, ICAM-1, VCAM-1 and CD44 [12–14]. Activated by metal ions, endothelial cells express these molecules in the lumen and attract granulocytes, macrophages and lymphocytes causing transmigration from blood to the tissue interface [15]. Metal particles and debris generated by wear in this region trigger macrophages to secrete pro-inflammatory cytokines, such as IL-8, IL-1beta, (IL-1β TNF-alpha, (TNF-α) and IL-6 and growth factors [16–18]. At a certain point in this milieu, MGCs and/ or osteoclasts are generated by fusion of macrophages [19] and a few of these cells get deposited on the metal surface of the implant. This results in progressive local osteolysis and leads to aseptic loosening, a major problem in patients with MoM hip implants [20]. Several studies show that cytokines influence the generation of osteoclasts: IFN-γ, IL-12 and IL-18 have all been shown to block the generation of osteoclasts [21–23], whereas IL-6 have been shown to promote osteoclast formation in rheumatoid arthritis [24].

To understand the process of osteolysis in physiologic conditions, there is a need for a suitable model to represent the relevant architecture of the tissue. The development of a tissue-like model is required to accurately determine the mechanism of osteoclast generation and their functions. Osteoclast generation requires 6–14 days of culturing primary mononuclear cells, with cytokine and growth factor treatment [19,25,26]. Previously, these experiments were performed in plastic or glass plates. However, the absence of a collagen matrix failed to provide spatial geometries to the cultured cells. This property is crucial in understanding the effect of metal particles and debris in a more realistic manner, as seen in tissue. The three-dimensional peripheral tissue equivalent (PTE) model comprised of collagen matrix with metal particles and debris, overlaid by endothelial cells and PBMCs, mimics the physiological environment of tissue near a metal implant. Besides a more realistic geometric spatial arrangement of cells, this model also provides an intercommunication system between the cells and the collagen matrix, which is a prerequisite for the generation of osteoclasts.

Several studies have shown endothelial cells facilitate osteoclast generation [27,28]. In biological studies with MoM hip implants, the role of endothelial cells in osteoclast generation has yet to be evaluated. The PTE model also serves as a sensitive system to evaluate endothelial cell function in the generation of osteoclasts in the presence of metal particles and debris. The PTE model was originally introduced by Randolph GJ *et*. *al*. to ascertain the generation of inflammatory cells from monocytes in a 3-D environment [29]. Therefore, we hypothesized that the PTE model would provide a tissue-like environment that would enhance the generation of osteoclasts by metal particles and debris compared to the traditional 2D cultures. For this purpose, we designed a PTE model with a modification consisting of collagen gel. Cobalt chromium alloy (CoCr ASTM F75, 1–5 micron) wear particles were added at the time of collagen gel polymerization. Endothelial cells were added to form a monolayer on the gel surface and human peripheral blood mononuclear cells (PBMCs) were seeded on top. The cultures were incubated for 14 days and harvested cells were analyzed for osteoclasts. We checked the expression of tartrate resistant alkaline phosphatase (TRAP) and dendritic cell specific transmembrane protein, (DC-STAMP), which are well known markers of osteoclasts [26]. Our results show that endothelial cells induced a greater number of osteoclasts in the 3-dimentional PTE system as compared to osteoclasts generated in the traditional 2-dimensional culture plates.

## Materials and Methods

### Human peripheral blood mononuclear cells preparation

Whole blood samples were obtained in sodium heparin treated tubes from the NIH blood bank (Bethesda, MD). The study was reviewed and approved by Institutional Review Board and Food and Drug Administration (IRB-FDA) and Research Involving Human Subjects Committee (RIHSC), IRB-FDA, RIHSC Protocol #12-050R (Silver spring, MD). All donors were in good health and blood samples were negative for blood-borne pathogens as detected by standard blood bank assays. The peripheral blood mononuclear cells (PBMCs) were enriched by Histopaque 1077 (Sigma, St. Louis, MO) according to standard laboratory procedures. Briefly, 25ml of whole blood was loaded on top of 10ml of histopaque 1077 (Sigma, St. Louis). The mix was centrifuged at 400g with brakes off and at room temperature for 30min. Interfacial cells were gently collected, washed with Hank’s balanced salt solution (HBSS) (Gibco, Green Island, NY) and resuspended in M199 medium (Sigma) with 10% heat inactivated AB serum (Sigma) supplemented with penicillin and streptomycin (Gibco). Cells were counted and adjusted to 5 x 10^6^ cells / ml.

### In vitro 3-dimensional (3-D) collagen model preparation

3-D collagen gels were prepared according to the manufacturer’s instructions. Briefly, 8 parts (600 μl) of collagen gel (Sigma) was added to one part of 10X Phosphate buffered saline (PBS) (75 μl) (Gibco) and neutralized with 67 μl of 0.1 N NaOH in a 1.5ml Eppendorf tube. 300 μl of this mixture was poured into wells of a 24 well plate (BD pharmingen, San Jose, CA) along with Co-alloy (A13) particles or lipopolysaccharide (LPS) (Sigma) and allowed to polymerize at 37°C. Co-alloy (A13) particles were purchased from BioEngineering Solutions Inc. (Chicago, IL; catalogue number: Co-A13, lot: 13974). A13 particles were prepared from total hip arthroplasty femoral head (Zim 40mm 12/4+7.0C 69124500) and provided at certified concentrations of 100x10^6^ particles/10μl in sterile PBS. The concentration of A13 particles in the gel was calculated with respect to the number of PBMCs, which were added at the beginning of the assay. For 100: 1 particle to cell ratio, 100 million particles were added to the gel for 1 million PBMCs, which were added two days later on the top of endothelial cell monolayer. All the gels were polymerized within 2h of incubation in a CO_2_ incubator. Following gel polymerization, EA Hy926 cells (ATCC, Manassas, VA) were added to form a monolayer on top over the next 48hr in a 5% CO_2_ incubator. Thereafter, the addition of PBMCs on top of the endothelial cell monolayer was considered as a starting point of the assay.

### PBMC—EA Hy926 co-culture

**Fig 1:**
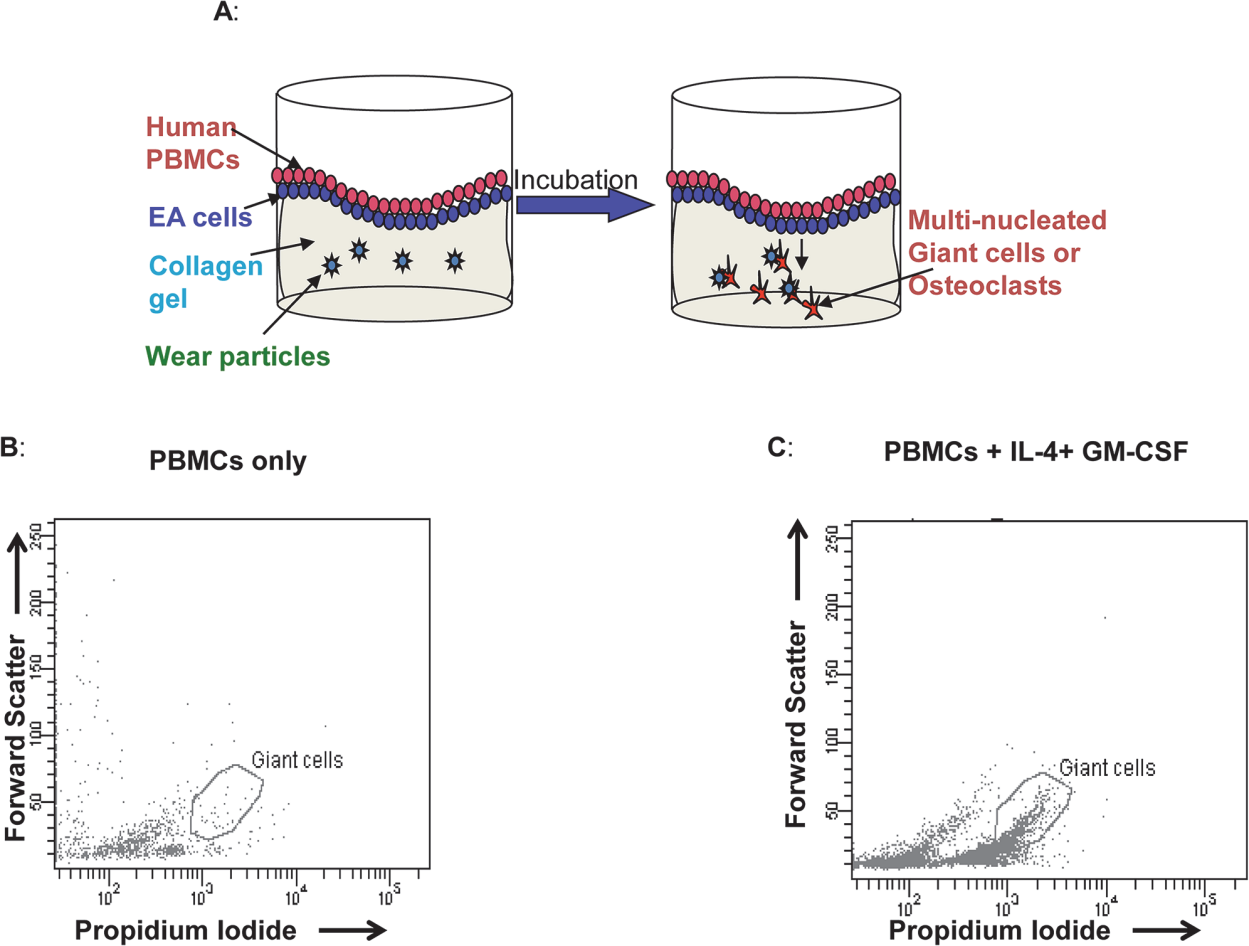
Development of Peripheral Tissue-Equivalent Model using collagen gel / EA Hy926/ PBMCs and characterization of MGCs by flow cytometers. A) For the 3-D peripheral tissue equivalent model, particles were added at the time of collagen gel polymerization. Endothelial cells (EA, EAHy926 cell line) were grown on the top of the collagen gel to form a monolayer. One million peripheral blood mononuclear cells (PBMCs) were seeded on top of the endothelial cell monolayer and incubated for 2 weeks. MGCs or OCs were characterized and quantified on flow cytometer. Collagen gels were polymerized as described in method One million peripheral blood mononuclear cells (PBMCs) were treated without (B) or with (C) cytokines IL-4 and GMCSF for two weeks. Cells were harvested by digesting the gel with Collagenase. Cells were washed, fixed and permeabilized using BD cytofix/ perm buffer, and stained with propidium iodide before acquisition on a flow cytometer.

To make the endothelial cell monolayer, 5 x 10^5^ EA Hy926 cells were layered on top of the collagen matrix in EA cell culture medium supplemented with 10% heat inactivated fetal bovine serum (FBS) (Gibco) and 50mg/ mL of endothelial growth factor (Lonza, Walkersville, MD) for 48h. The monolayer was gently washed with 1X PBS before adding PBMCs. At the start of the assay, 1.0 x 10^6^ PBMCs were suspended in M199 media containing 10% heat inactivated AB serum, and applied onto the EA monolayer at 10^6^ cells/ml/ well, as described in Fig 1A. In selected experiments, recombinant human IL-4 (20ng/mL, R&D Systems, Minneapolis, MN, USA) and recombinant human GM-CSF (20ng/mL, R&D Systems) were added to cell cultures for generation of multinucleated giant cells (MGCs). Cells were incubated for 14 days without changing the medium. At 4^th^, 7^th^, 9^th^ and 14^th^ day, supernatants were collected and centrifuged before storing in -80°C freezers. Migrated cells inside the gel matrix were collected by digesting the gel with 5μg/ 100μl collagenase D (Roche, NJ) in HBSS for 3 min in a CO_2_ incubator.

### Staining of multinucleated giant cells and osteoclasts

The number and phenotype of MGCs was determined using flow cytometry as performed previously [30]. Cells were harvested following collagenase digestion of the gels for 5 min. Cells were washed in HBSS and then treated with BD Biosciences fixation buffer for 20 min. Cells were re-washed and incubated with BD’s permeabilization buffer for 30 min. Then, the cells were treated with propidium iodide (PI, 3μl/test) (BD Pharmingen) for 20 min in FACS buffer, which was freshly made, 0.22μ filtered using EMD Millipore Stericup (Fisher Scientific, Waltman, MA) and composed of 3% heat inactivated FBS in HBSS. After intracellular staining, cells were washed twice with FACS buffer. For oesteoclast characterization, the permeabilized cells were stained with Fluorescein Isothiocyanate (FITC)-conjugated Tartrate resistant acid phosphatase (TRAP) (Biorbyt, San Francisco, CA) and Allophycocyanin conjugated Dendritic cell specific trans membrane protein (DC-STAMP) (R& D Systems, Minneapolis, MN) for 30 min, washed twice in FACS buffer and finally suspended in 500μl of FACS buffer before acquisition on the BD canto II flow cytometer.

### Flow cytometry

The number and phenoytpe of MGCs and osteoclasts were determined using a flow cytometry method as performed previously [30]. Briefly, the gating strategy was plotted using a dot plot for forward scatter (FSC) and PI signal on a FACSDiva. An increase in cell volume and multinucleated cells were determined by FSC in linear scale and PI signal in log scale respectively (Fig 1B). A gate for double positive cells denotes MGCs. A total of 10,000 or 50,000 events were acquired for each sample. For osteoclast quantification, PI positive cells were further analyzed for TRAP and DC-STAMP. Thus, osteoclasts are characterized by large cell volume and are positive for PI, TRAP and DC-STAMP. The experiments were perfomed individually three times for “0:1” and “10:1” as indicated in the figure. High ratios of particles to cell “500:1” and “100:1” was perfomed once (as at this ratio most of cells were considered to be dead by 14 days to show the effect).

### Luminex assay

Cell supernatants from 3D culture were analyzed for cytokine production using the Inflammatory Cytokine Human 5-Plex Panel (Invitrogen, Camarillo, CA) and human /monkey extracellular protein buffer reagent kit (Invitrogen). Supernatants were harvested from three independent experiments, n = 3. Assays were run on the Luminex 200 platform (Luminex, Corporation Austin, TX), following the manufacturer’s instructions. Individual samples were run in triplicate and the MasterPlex 2010 (Hitachi Solutions America, Ltd., San Francisco, CA) best-fit logistic curve-fitting method was used to calculate IL-2, IL-4, IL-10, IFN-γ, and IL-6. Cytokine standard curves were generated from the means of duplicate measurements, while internal controls and experimental samples were measured in triplicate. (The sensitivity of the assay was at 5pg/ml)

### Live/Dead cell viability assay

LIVE/DEAD Viability kit was purchased from Life Technologies (Grand Island, NY). As per manufacturer’s protocol, 50 γl of DMSO from vial labeled B was thoroughly mixed with Dye in vial labeled A and kept in room temperature for 15 min. Cells from particle treated co-cultures were harvested from 3D collagen gel by collagenase treatment and from conventional 24 well plates by gentle pipetting. Approximately 1 x 10^6^ cells were washed twice with HBSS and reconstituted in final 1mL of HBSS again. 1 μl of reconstituted viability dye was added to the cells and incubated for 30 min. Cells were washed twice with HBSS to remove unbound or excess dye. Finally, cells were transferred in 500 μL of HBSS before acquiring on the BD Canto II. 50,000 events were acquired for each test, the lymphocyte region was gated on forward scatter versus side scatter dot plot, the dead cell gate was drawn using unstained control “cells without viability dye” and frequency of dead cells were obtained as indicated in “dead cell dye gate”.

### RT-PCR

Total RNA from Co-alloy particle-treated and untreated cultures was isolated and purified using the RNeasy Plus Mini Kit (Qiagen Technologies, Gaithersburg, MD). RNA concentration was determined using a Qubit 2.0 Fluorometer (Life Technologies, Grand Island, NY). The total RNA was collected from two independent experiments, n = 2. RNA (0.5–1 μg) was reverse transcribed into cDNA using the QuantiTect Reverse Transcription Kit (Qiagen) as per manufacturer’s instructions. Two-Step real-time PCR was carried out by amplifying diluted cDNA (< 100 ng/reaction) using the QuantiFast SYBR Green PCR Kit (Qiagen) and the following gene specific primers (Invitrogen): 5′-AGAGCTGCAGTACCGATTGG-3′ (forward) and 5′-GTAGAAGGCTGCGATGACCCT-3′ (reverse) for DC-STAMP; 5′-CCTGGTACGTGCTAGCCG-3′ (forward) and 5′-TCTTGAAGTGCAGGCGGTAG-3′ (reverse) for TRAP; 5′-AGCCGAACCTGCCTACAGGAC-3′ (forward) and 5′-GGGCAGTGCTGCTTGTAGGTG-3′ (reverse) for GM-CSF; and 5′-CCAGGTGGTCTCCCTCTGACTTC-3′ (forward) and 5′-CACCCTGTTGCTGTAGCCAAA-3′ (reverse) for GAPDH. The PCR reaction mix was run on the LightCycler 480 II Instrument (Roche Molecular Biochemicals, Indianapolis, IN) and the following PCR run protocol was used: 95° C, 5 min (denaturation); 40–45 amplification cycles of 95° C for 10 s, 60° C for 10 s and 72° C for 20 s, followed by melting curve analysis to ensure the specificity of PCR amplification. GAPDH was used as the reference gene for every target gene per sample, and data were normalized against the respective GAPDH signal. Cycle threshold (C_T_) values were determined using the LightCycler 480 II software (Roche Molecular Biochemicals, version 1.5). Relative transcript expression values were obtained by normalizing C_T_ values of the target genes with C_T_ values of the housekeeping gene copy number using the 2^−ΔΔCt^ method.

### Statistical analysis

All data were expressed as mean + S.E. Individual experiments were performed a minimum of three times (n > 3). The software package Instat 2 (Graph Pad Prism, version 5) (Graph Pad Software, San Diego, CA) was used for statistical analysis of data. Significant differences between groups were determined with student’s t-test or one-way analysis of variance (one-way ANOVA) followed by Dunnett’s or Bonferroni’s post-hoc test to compare significance of differences between means. A p-value< 0.05 was considered significant.

## Results

### Development of Peripheral Tissue-Equivalent Model and characterization of MGCs

Collagen gels were prepared as described in materials and methods. Freshly isolated PBMCs, 1x10^6^ were seeded on top of the collagen gel surface in the presence of IL-4 and GM-CSF, (both at 20ng/ml) (Fig 1A). Cultures were incubated for 14 days without changing the medium. Cells were harvested, fixed, permeabilized and stained with PI as described in methods. Results showed that the combination of IL-4 and GM-CSF induced MGC formation, shown by a polygonal gate which specifies cells with high forward scatter and PI intensity (Fig 1C), compared to a similar polygonal gate in untreated cells, Fig 1B.

### Three dimensional collagen gels support the development of MGCs

**Fig 2:**
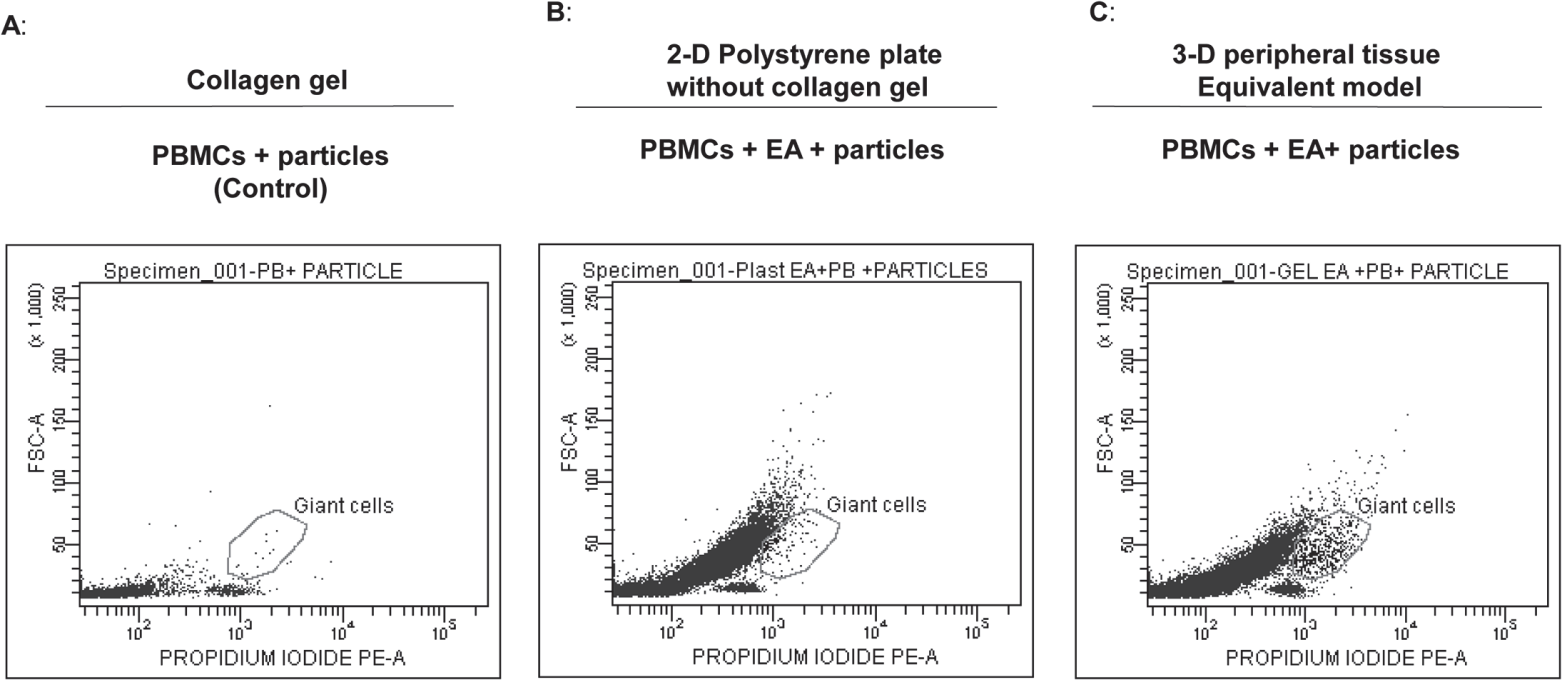
3-D model containing collagen gel generates a greater number of MGCs. For the 3D model, 100 million particles were added at the time of gel polymerization (a ratio of 100:1 particles: PBMCs) and a monolayer of endothelial cells was grown on top of the gel (C). For the 2D model, endothelial cells were grown to form a monolayer and the particles were added to the monolayer (B). One million PBMCs were seeded on top of the endothelial monolayer in both models and incubated for 2 weeks. As a control, PBMCs alone were exposed to gel embedded with particles (A). On the 14th day, cells were harvested (following collagenase treatment in the 3D model), fixed and permeabilized using BD cytofix/perm buffer. Cells were then stained with PI and acquired on a flow cytometer.

First, 10x 10^6^ A13 particles were polymerized with the collagen gel to form the 3D structure. A similar number of A13 particles were added directly to wells of 24- well polystyrene plates. The endothelial cell monolayer was added on to the surface of the particle-embedded collagen gel or directly on plastic plates with particles. 1x 10^6^ PBMCs in total volume of 1ml complete medium were seeded on top of the endothelial cell monolayer and incubated for 14 days. Cells were carefully harvested and stained for MGCs. As shown in Fig 2C, the 3D collagen gel demonstrates higher generation of MGCs as compared to co-culture without collagen gel, Fig 2B, and control PBMCs alone on gel, Fig 2A.

### Increase in ratio of A13 particles to PBMCs does not enhance MGC formation

**Fig 3:**
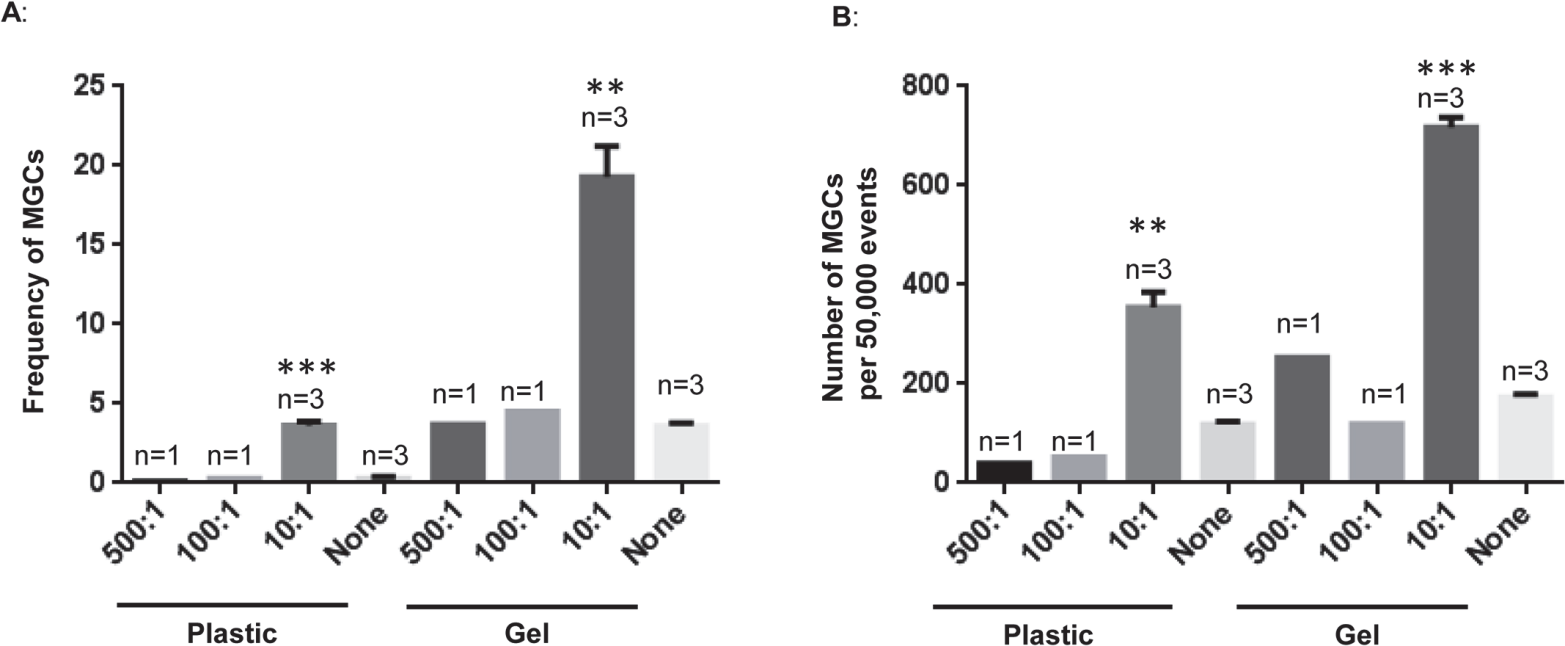
Higher frequency and number of MGC formation at a 10:1 ratio compared to 100:1 and 500:1 ratios in 3-D model. Particles were added at the time of gel polymerization at a ratio of 500:1, 100:1, or 10:1 or without particles (0:1) to PBMCs, as described previously, and incubated for 2 weeks. Cells were harvested with collagenase treatment, fixed and permeabilized using BD cytofix/ perm buffer, and stained with propidium iodide before acquisition on a flow cytometer. Frequency of MGCs per 50,000 events is represented in A, while the MGCs number is represented in B. The results are represented as mean +/- S.E. of three independent experiments, shown as n = 3 for “0:1” and “10:1”. Asterisks indicate statistically significant MGCs increase in particles exposed cells at 10:1 compared with those untreated cells, none. (** = p < 0.005; *** = p <0.0005).

In order to examine the effect of a higher ratio of A13 particles to PBMC, on MGC formation, co-cultures were set up at ratios of 500:1, 100:1 and 10:1, and compared to co-cultures without A13 particles (0:1). The cultures were established in 24-well plates. PBMCs were harvested after 14 days for measurement of MGC formation using flow cytometry. As expected, more MGCs were generated with particle-embedded—collagen gel than with particles in the absence of collagen gel. Without a collagen matrix the frequency of MGCs at 10:1 ratio was 4% compared to 0.2% in cells without particles, (Fig 3A). MGCs were at 0.2% at 500:1 and 100:1 ratios. In the presence of collagen, the frequency of MGCs at 10:1 ratio was 20% compared to 4% in cells without particles. Ratios of 500:1 and 100:1 did not enhance the frequency of MGC formation beyond 4%, (Fig 3A). A similar trend was observed when the number of MGCs per 50,000 events was analyzed. In the absence of collagen gel, the number of MGCs was around 380 at 10:1 ratio compared to 170 in untreated cells, Fig 3B. MGCs numbers were around 70 at 100:1 and 50:1 ratio. A collagen gel-dependent culture induced higher numbers of MGCs at a 10:1 ratio compared to cells with no particles (780 MGCs versus 180 MGCs respectively). MGC numbers were around 200 for 500:1 and 100 for 100:1 ratios, Fig 3B.

### IFN-γ was reduced while IL-10 was increased in the 10:1 (particles: PMBC) treatment supernatants of day 14 cultures

**Fig 4:**
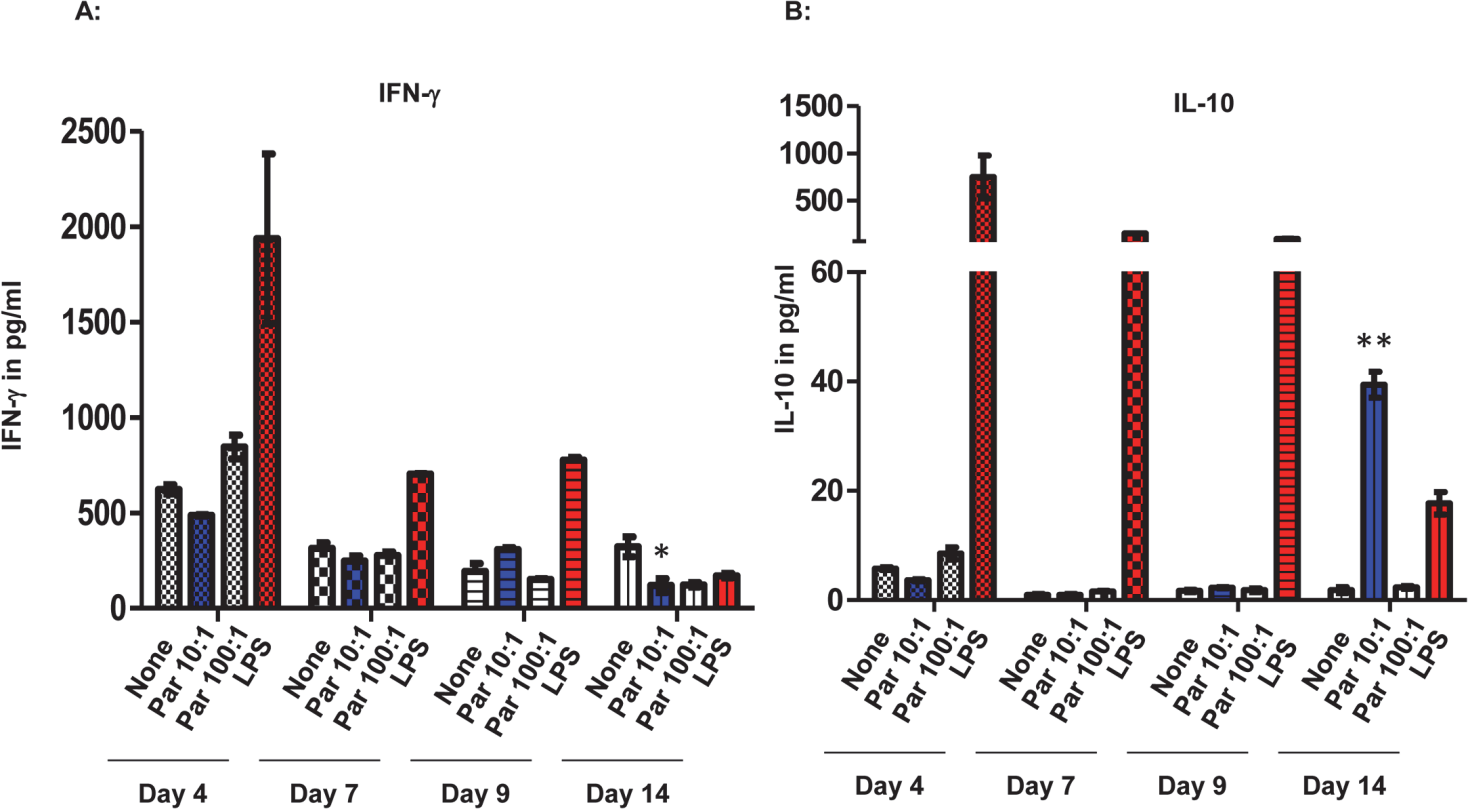
The level of IFN-γ was reduced while IL-10 level was increased in 10:1 treatment supernatants of day 14 culture. The 3-D model was prepared as previously stated, and supernatants were collected on days 4, 7, 9, and 14. Luminex Cytokine Th1/Th2 5-plex Immunoassay kit was used to measure the concentrations of IFN-γ, IL-2, IL-4, IL-5, and IL-10. The results are represented as mean +/- S.E. of three experimental samples from three independent experiments, n = 3. Asterisks, * = p < 0.05, indicate statistically significant IFN-γ decrease in 10:1 particles exposed cells at day 14 compared with those at day 4. ** = p < 0.005 indicate statistically significant IL-10 increase in 10:1 particles exposed cells at day 14 compared with those at day 4.

The 3-D model was prepared as previously stated, and supernatants were collected on days 4, 7, 9, and 14. We measured the concentrations of IFN-γ, IL-2, IL-4, IL-5, and IL-10. At the 10:1 A13 particles to PBMC ratio, the IFN-γ concentration was 500pg/mL ± 15pg/mL in the supernatants of day-4 cultures. There was a decrease in the concentration of IFN-γ, to 80pg/ml ± 5pg/ml in the day 14 culture supernatants, (Fig 4A). Conversely, IL-10 levels from the same cultures were below the level of detection (< 4pg/ml) on day 4, and rose to 41pg/ml +/- 2pg/ml by day 14, (Fig 4B). IL-2, IL-4 and IL-5 levels did not change significantly in cultures at different time period.

### Elevated mRNA levels of TRAP, DC-STAMP and GM-CSF at ratio of 10:1 in day 14 co-cultures

**Fig 5:**
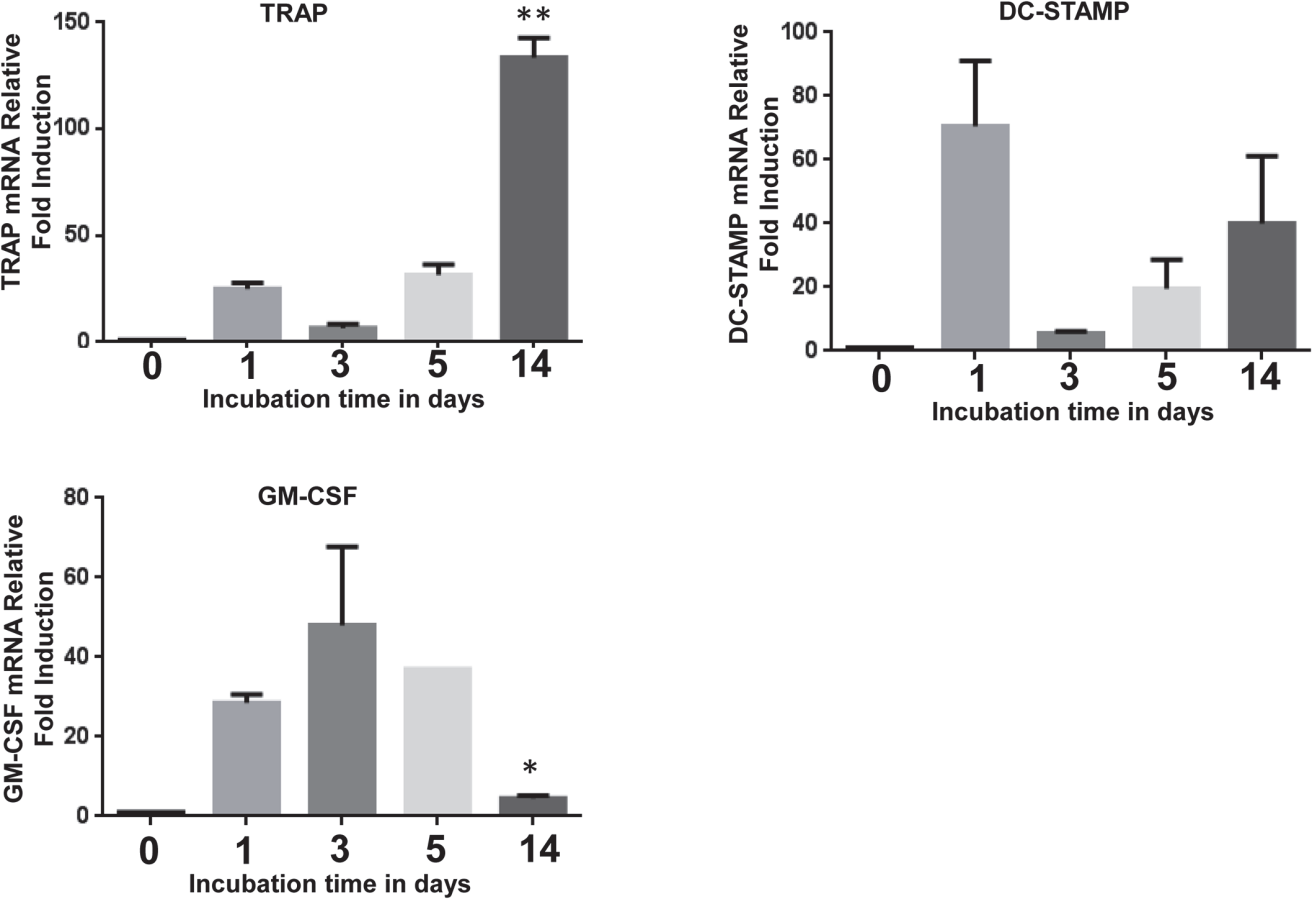
Elevated mRNA levels of TRAP, DC-STAMP and GM-CSF in day 14 co-cultures. Particles were added at the time of gel polymerization at the ratio of 10:1 particles to PBMCs. Endothelial cells (EA) were grown on the gel to form a monolayer. Peripheral blood mononuclear cells (PBMCs) were seeded either on top of the monolayer. Cells were harvested by digesting the gel with RNA extraction buffer and proceeded for RT-PCR as described in method. Each bar is represented as mean +/- S.E. of two experimental samples from two independent experiments, n = 2. *Asterisks indicate statistically significant TRAP and GM-CSF increase in particles exposed cells at day 14 compared with day0, (** = p< 0.005, * = p < 0.05)

Total RNA was harvested from the cells at days 0, 1, 3, 5 and 14 and performed real time qPCR to quantify the markers of osteoclast, TRAP and DC-STAMP. Granulocyte macrophage colony stimulating factor (GM-CSF) is an inducer of MGCs and was also measured in this assay. The results showed that A13 particles enhanced the expression levels of TRAP and GM-CSF to 140 fold and 8 fold, respectively, compared to culture controls at day 0. Although the mRNA levels for GM-CSF did not increase significantly, we observed a trend in the up-regulation of GM-CSF transcript levels, Fig 5.

### A13 particles up-regulated TRAP and DC-STAMP expression on MGCs

**Fig 6:**
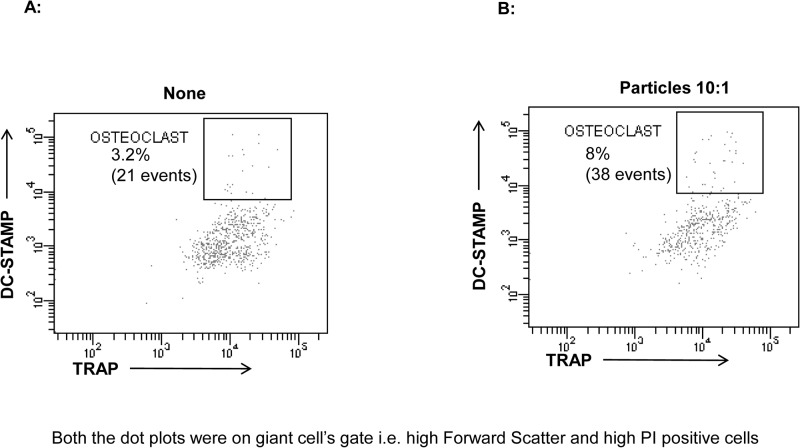
Particles at 10:1 ratio induced protein expression the DC-STAMP and TRAP in day 14 cultures. As described above, co-cultures were set up at 10:1 ratio and incubated for 14 days. Cells were harvested by collagenase treatment, washed and intracellular stained with propidium iodide, FITC conjugated- TRAP and APC conjugated-DC-STAMP. Both the dot plots (A and B) were first gated for giant cell i.e. high Forward Scatter and high PI positive cells, and then for DC-STAMP and TRAP. Cells without particles are represented in (A) and with particles in (B).

To examine the differentiation of MGCs into osteoclasts, the co-culture was set up on collagen gel in the presence of a particle-to-cell ratio of 10:1 and cells were harvested for evaluation of DC-STAMP and TRAP, the markers for osteoclasts. Cells were gated for high forward scatter and PI positive cells, i.e. giant cells, and further analyzed for osteoclast markers. Particle exposed cells showed a frequency of double positive expression of DC-STAMP and TRAP of 9% compared to 4.1% in cells without particles, (Fig 6).

## Discussion

A13 particle-induced osteolysis, aseptic loosening and pseudo-tumor formation are the major concerns in a number of MoM bearing patients diagnosed with pain and limited motion of the joint(s) [8]. With increasing demand for total hip arthroplasty, improvements in its mechanical strength and design, the regulatory requirements for early diagnosis of such adverse events and immune-toxicity testing are essential to improve the quality of life of MoM bearing patients. A simple, economic and representative *in vitro* model similar to peripheral tissue around the implant site can provide valuable information regarding periprosthetic tissue reactions against debris and particles, which leads to MGC formation and osteoclast generation. MGCs and osteoclasts migrate and cover bone surfaces around the implant site and initiate bone degradation. The biomolecules, which appear in serum during the generation of these cells, could be potential biomarker candidates. The release of inflammatory cytokines is associated with synthesis of short RNA sequences called micro RNA, which regulate gene expression. This microRNA can also serve as a potential biomarker, which may be evaluated using this model. Our 3-dimensional *in vitro* model provides a tissue like environment for the generation of MGCs and osteoclasts *in situ*. The use of the PTE model to evaluate per prosthetic tissue reaction of immune cells to particles has been introduced in our study with a motive to help in the diagnosis and to understand the molecular mechanism of OC generation. Importantly, using this model we found that endothelial cells enhance MGCs and osteoclast generation in the presence of A13 particles.

**Fig 7:**
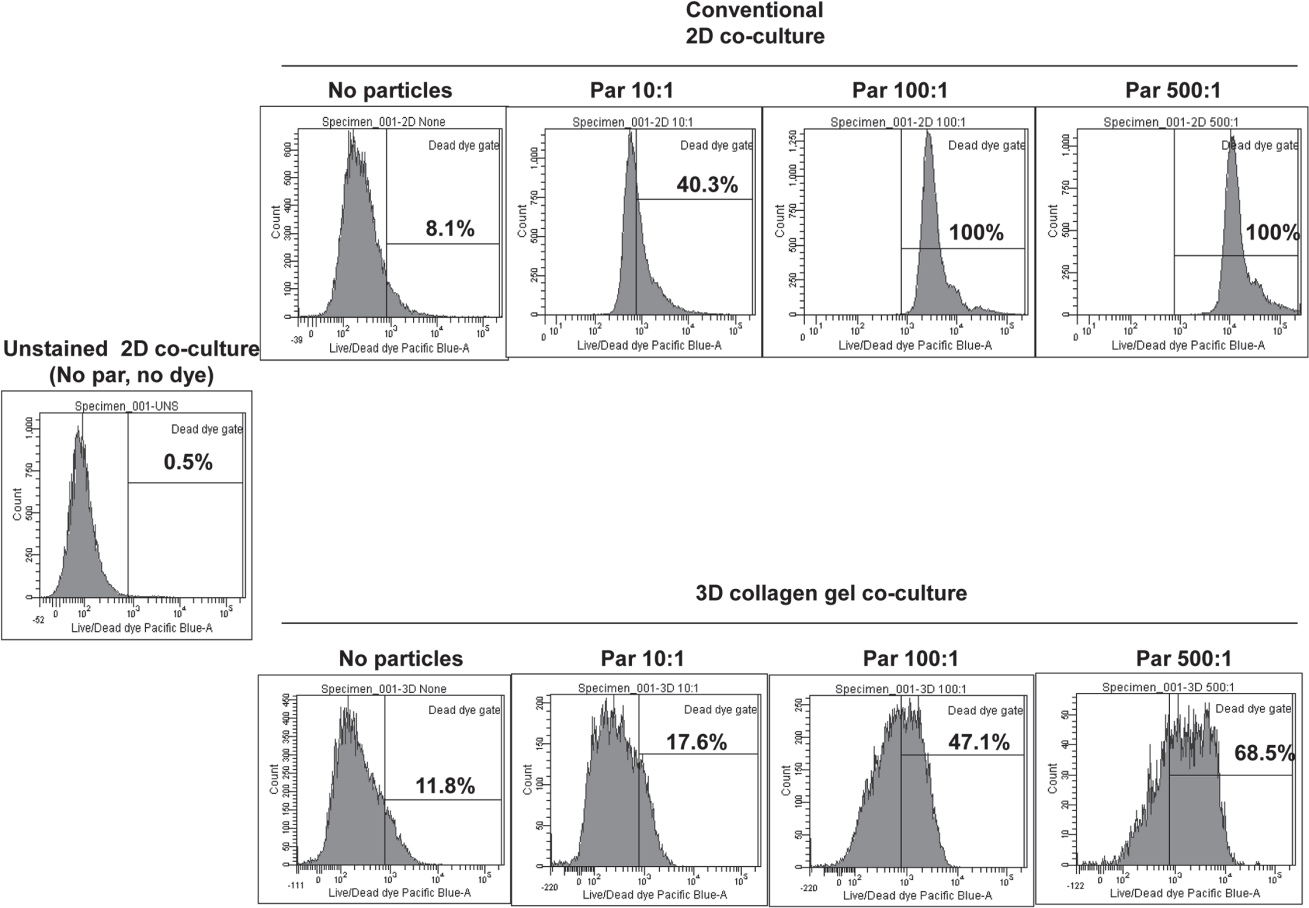
Increase in particle to cell ratio increases frequency of dead cells. Particles were added at the time of gel polymerization at a ratio of 500:1, 100:1, or 10:1 or without particles (0:1) to PBMCs, as described previously, and incubated for 48h. Similar particle treated co-culture was set up in the absence of collagen gel in conventional 24 well plate. Cells were harvested with collagenase treatment from collagen gel, and stained with Live/Dead dye before acquisition on a flow cytometer. Cells from conventional culture plates were harvested by gentle pipetting and stained with Live/ Dead dye. 50,000 events were acquired on BD canto II and frequency of dead dye positive cells is represented in figure.

The addition of a third dimension to the cell environment provides more surface area for cell adhesion, which is necessary for integrin ligation, cell contraction and even intracellular signaling [31]. The gene expression profile of cells grown in a 3D environment is different from those cells grown in 2D plastic or glass dishes [32]. As shown in Fig 2, we observed enhanced generation of MGCs in a 3D model compared to plastic plates. These results indicate that the cell-to-cell and cell-to-collagen interaction is a requisite for expression of biomolecules, which are necessary for MGC formation. An increase in the particle-to-cell ratio to 500:1 and 100:1 might have triggered apoptosis in the co-cultures and hence the 10:1 particle to cell ratio could possibly provide optimal conditions required for OC generation. As shown in Fig 7, an increase in particle to cell ratio induced more cell death for both the 2D and 3D collagen gel cultures. However the 3D collagen gel cultures were more resistant to cell death than the 2D culture system. For example, a 100:1 particle to cell ratio in the 2D system resulted in 100% cell death, whereas the 3D collagen gel cultures at the same ratio yielded 47% cell death. The reduced cell death effect might be due to dispersion of particles in 3D gel and thus few of the cells might have find a space (living environment) in the gel and in between the particles. In MoM bearing tissue, these non-apoptotic cells may directly or indirectly activated by pro-inflammatory cytokines, thus recruiting more lymphocytes. The activation and differentiation of non-apoptotic cells may lead to pseudo tumor like tissues as observed in MoM bearing patients with chronic pain. Molecular characterization of the giant cells in collagen gel will provide the important clues in our understanding of the generation of osteoclasts and pseudo tumor formation. The addition of IL-4 and GM-CSF induces the generation of MGCs from peripheral blood cells [19]. We measured IL-2, 4, -5, 10 and IFN- γ in the supernatants from 3D culture at different time intervals. IFN- γ levels in the supernatant were higher in early time points (day 4, 7 and 9) than at day 14. Conversely, IL-10 levels were lower in early time points than at day 14, which suggests a shift from a Th1 response to Th2 over time and we detected MGCs when IFN- γ levels were low. This is in accordance with the findings of Kohara H *et*. *al*. with regards to inhibition of multinucleated cell formation by IFN- γ [22].

Endothelial cells line the inner wall of blood vessels and act as a barrier between white blood cells and surrounding tissues. These cells are the reservoirs of immune-modulatory molecules like cytokines, chemokines and adhesion molecules and are responsive to foreign antigens. Upon sensing a danger signal, endothelial cells upregulate adhesion molecules to promote infiltration of inflammatory cells such as monocytes, neutrophils, etc., from blood vessels to tissues where foreign antigens are present. Further, endothelial cell derived cytokines and other bioactive molecules facilitate anti- foreign body tissue responses. A similar cellular infiltration and cytokine burst processes are mediated by endothelial cells in the presence of A13 particles and ions [12–15]. We developed a 3D peripheral tissue equivalent model consisting of collagen gel with embedded A13 particles, an endothelial cell monolayer on the gel surface and peripheral blood mononuclear cells on top, which resembles tissue surrounding a prosthesis. We found that endothelial cells significantly enhance the generation of MGCs. Such an *in vitro* model can be a benefit in initial screening of drugs that can intervene in MGC generation. Potential biomarkers to predict the failure of orthopedic devices can also be determined and followed by their evaluation in clinical samples from hip bearing patients with osteolysis.

OCs and osteoclast like cells are bone-resorbing, multinucleated cells. OCs are generated in the presence of a membrane fusogen transmembrane protein called dendritic cell specific transmembrane protein (DC-STAMP)[33]. DC-STAMP induces cell fusion of mononuclear precursor cells to form multinucleated OCs, which plays a critical role of bone destruction in Rheumatoid arthritis [34,35]. Tartrate-resistant acid phosphatase (TRAP) is also expressed by osteoclasts and has a critical role in many biological processes including skeletal development, collagen synthesis and degradation, the mineralization of bone, and in immune-modulatory function [36,37]. Both DC-STAMP and TRAP are considered biomarkers of OC [26,38]. We measured the expression level of these OC biomarkers and found a significant increase in their expression on day 14 cultures as shown in Fig 5. Thus, we successfully developed a 3D peripheral tissue equivalent model and showed that A13 particles embedded in collagen gel can trigger the generation of OC from peripheral blood mononuclear cells, and endothelial cells play a vital supporting role in OC formation.

## Disclaimer

The mention of commercial products, their sources, or their use in connection with material reported herein is not to be construed as either an actual or implied endorsement of such products by the Department of Health and Human Services. The findings and conclusions in this article have not been formally disseminated by the U.S. Food and Drug Administration and should not be construed to represent any Agency determination or policy.

## Supporting Information

S1 Fig IL-4 and GM-CSF induces multinucleated giant cell formation in 3D-system. Collagen gels were polymerized as described in method One million peripheral blood mononuclear cells (PBMCs) were treated without or with cytokines IL-4 and GMCSF for two weeks. Cells were harvested by digesting the gel with Collagenase. Cells were washed, fixed and permeabilized using BD cytofix/ perm buffer, and stained with propidium iodide before acquisition on a flow cytometer. The gating strategy is shown in this figure.
(PDF)

S2 Fig Three-dimensional system induces more giant cell than two-dimensional system. S Particles were added at time of gel polymerization, endothelial cells were grown to form a monolayer and PBMCs were added on top as described in method section. Co-cultured cells were incubated for two weeks in 3D system. Particles treated PBMCs were added on top of endothelial cell monolayer grown directly on conventional polystyrene 24 well plates. Harvested cells were stained with PI and acquired on flow cytometer.
(PDF)

S3 Fig Higher MGC formation at a 10:1 ratio compared to 100:1 and 500:1 ratios. Particles were added at the time of gel polymerization at a ratio of 500:1, 100:1, or 10:1 or without particles (0:1) to PBMCs, as described previously, and incubated for 2 weeks. Cells were harvested with collagenase treatment, fixed and permeabilized using BD cytofix/ perm buffer, and stained with propidium iodide before acquisition on a flow cytometer.
(XLSX)

S4 Fig IFN- γ and IL-10 levels in 10:1 treatment supernatants of day 14 culture. The 3-D model was prepared as previously stated, and supernatants were collected on days 4, 7, 9, and 14. Luminex Cytokine Th1/Th2 5-plex Immunoassay kit was used to measure the concentrations of IFN- γ, IL-2, IL-4, IL-5, and IL-10.
(PDF)

S5 Fig Elevated mRNA levels of TRAP, DC-STAMP and GM-CSF in day 14 cultures. Particles were added at the time of gel polymerization at the ratio of 10:1 particles to PBMCs. Endothelial cells (EA) were grown on the gel to form a monolayer. Peripheral blood mononuclear cells (PBMCs) were seeded either on top of the monolayer. Cells were harvested by digesting the gel with RNA extraction buffer and proceed for RT-PCR as described in method. Data set is provided from two independent experiments.
(PDF)

S6 Fig Particles at 10:1 ratio induced expression of DC-STAMP and TRAP. As described above, co-cultures were set up at 10:1 ratio and incubated for 14 days. Cells were harvested by collagenase treatment, washed and intracellular stained with propidium iodide, FITC conjugated- TRAP and APC conjugated-DC-STAMP. The gating strategy is shown in this figure.
(PDF)

S7 Fig Increase in particle to cell ratio increases frequency of dead cells. Particles were added at the time of gel polymerization at a ratio of 500:1, 100:1, or 10:1 or without particles (0:1) to PBMCs, as described previously, and incubated for 48h. Similar particle treated co-culture was set up in the absence of collagen gel in conventional 24 well plate. Cells were harvested with collagenase treatment from collagen gel, and stained with Live/Dead dye before acquisition on a flow cytometer.
(PDF)
