# Supplementary material for: Wear Particles Derived from Metal Hip Implants Induce the Generation of Multinucleated Giant Cells in a 3-Dimensional Peripheral Tissue-Equivalent Model
Source: PLoS One. 2015 Apr 20;10(4):e0124389. doi: 10.1371/journal.pone.0124389 (PMC4403993; doi:10.1371/journal.pone.0124389)
Supplement: S1 Fig — Collagen gels were polymerized as described in method One million peripheral blood mononuclear cells (PBMCs) were treated without or with cytokines IL-4 and GMCSF for two weeks. Cells were harvested by digesting the gel with Collagenase. Cells were washed, fixed and permeabilized using BD cytofix/ perm buffer, and stained with propidium iodide before acquisition on a flow cytometer. The gating strategy is shown in this figure. (PDF) [file pone.0124389.s001.pdf]

FACSDiva Version 6.1.3

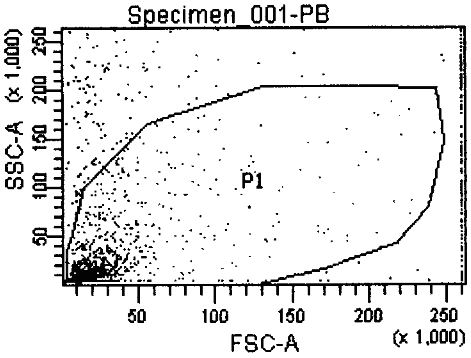

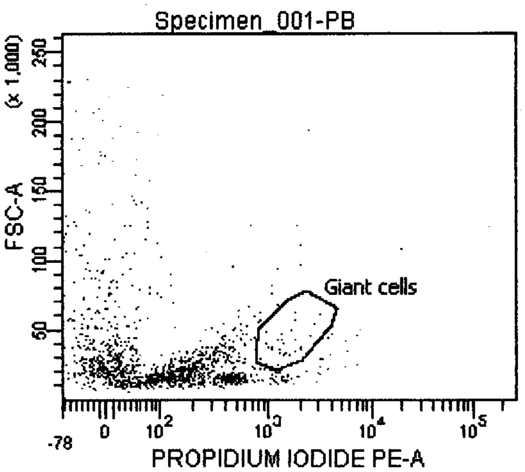

Tube: PB

Population	#Events	%Parent	%Total
All Events	3,267	####	100.0
P1	1,347	41.2	41.2
Giant cells	29	2.2	0.9

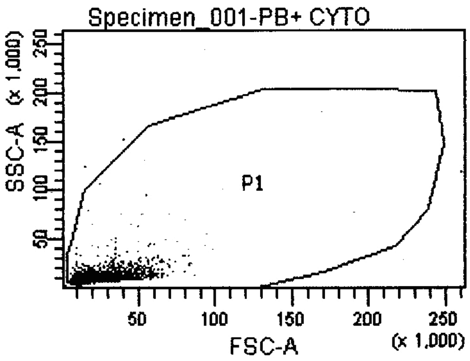

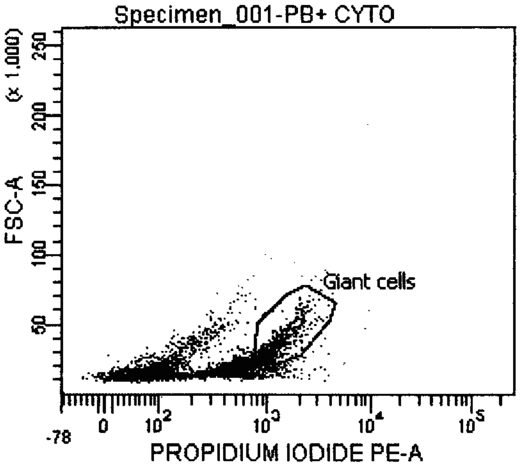

Tube: PB+ CYTO

Population	#Events	%Parent	%Total
All Events	20,277	####	100.0
P1	13,226	65.2	65.2
Giant cells	713	5.4	3.5
