# Supplementary material for: Wear Particles Derived from Metal Hip Implants Induce the Generation of Multinucleated Giant Cells in a 3-Dimensional Peripheral Tissue-Equivalent Model
Source: PLoS One. 2015 Apr 20;10(4):e0124389. doi: 10.1371/journal.pone.0124389 (PMC4403993; doi:10.1371/journal.pone.0124389)
Supplement: S2 Fig — S Particles were added at time of gel polymerization, endothelial cells were grown to form a monolayer and PBMCs were added on top as described in method section. Co-cultured cells were incubated for two weeks in 3D system. Particles treated PBMCs were added on top of endothelial cell monolayer grown directly on conventional polystyrene 24 well plates. Harvested cells were stained with PI and acquired on flow cytometer. (PDF) [file pone.0124389.s002.pdf]

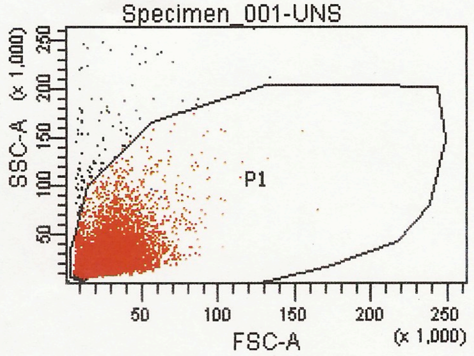

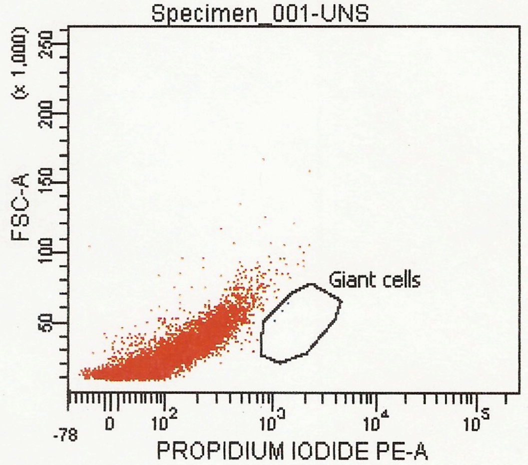

Tube: UNS

Population	#Events	%Parent	%Total
All Events	10,611	####	100.0
P1	9,292	87.6	87.6
Giant cells	3	0.0	0.0

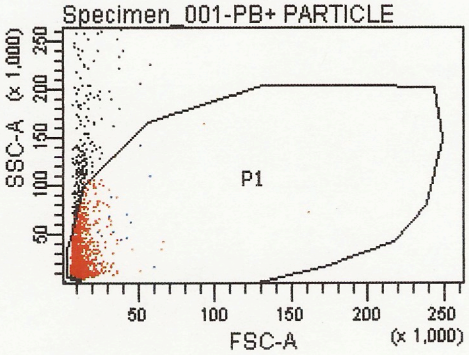

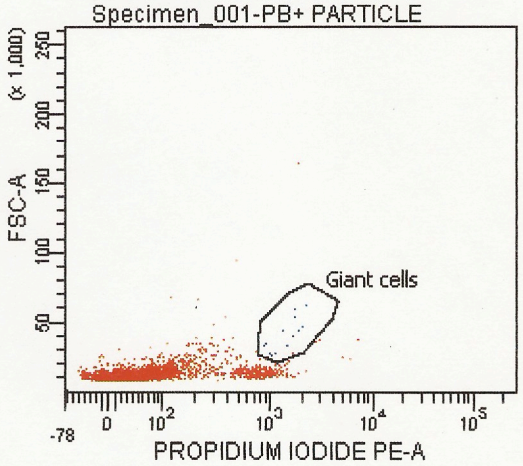

Tube: PB+ PARTICLE

Population	#Events	%Parent	%Total
All Events	5,211	####	100.0
P1	2,439	46.8	46.8
Giant cells	13	0.5	0.2

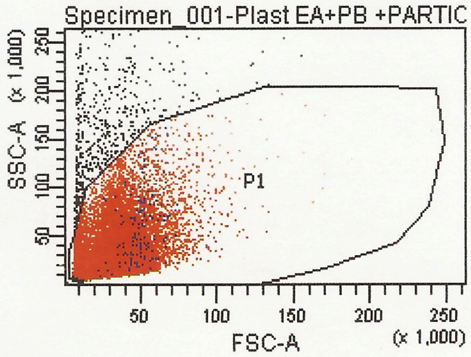

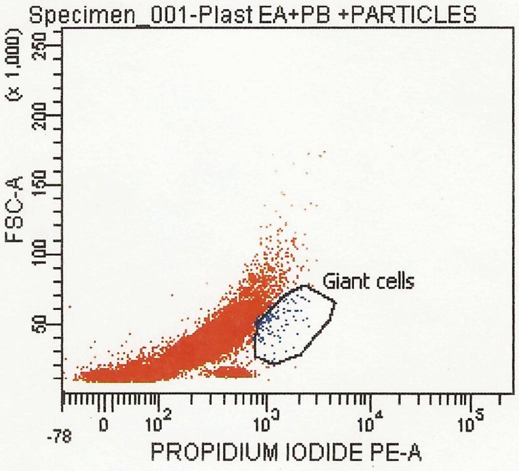

Tube: Plast EA+PB +PARTICLES

Population	#Events	%Parent	%Total
All Events	22,518	####	100.0
P1	19,908	88.4	88.4
Giant cells	156	0.8	0.7

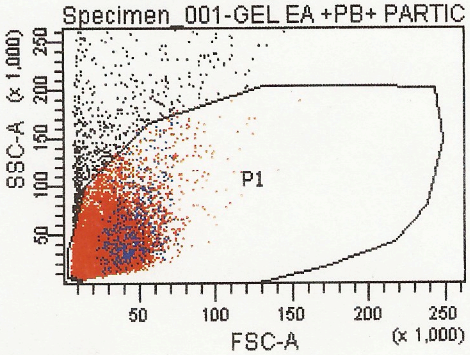

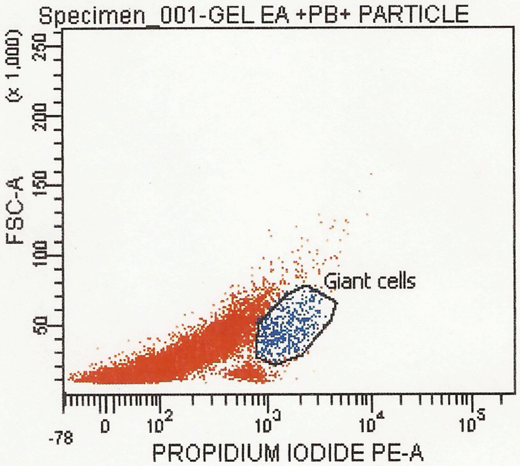

Tube: GEL EA +PB+ PARTICLE

Population	#Events	%Parent	%Total
All Events	24,354	####	100.0
P1	21,530	88.4	88.4
Giant cells	509	2.4	2.1
