# Supplementary material for: Wear Particles Derived from Metal Hip Implants Induce the Generation of Multinucleated Giant Cells in a 3-Dimensional Peripheral Tissue-Equivalent Model
Source: PLoS One. 2015 Apr 20;10(4):e0124389. doi: 10.1371/journal.pone.0124389 (PMC4403993; doi:10.1371/journal.pone.0124389)
Supplement: S4 Fig — The 3-D model was prepared as previously stated, and supernatants were collected on days 4, 7, 9, and 14. Luminex Cytokine Th1/Th2 5-plex Immunoassay kit was used to measure the concentrations of IFN- γ, IL-2, IL-4, IL-5, and IL-10. (PDF) [file pone.0124389.s004.pdf]

| | IFN-y [pg/mL] | | |
|---|---|---|---|
| Day 4 | 1 | 2 | 3 |
| None | 638.25 | 576.03 | 653.32 |
| 10 to 1 | 483.45 | 480.81 | 495.92 |
| 100 to 1 | 734.91 | 846.46 | 953.29 |
| LPS | 1516.8 | 1464.94 | 2825.01 |
| | | | |
| Day 7 | 1 | 2 | 3 |
| None | 370.67 | 282.26 | 289.89 |
| 10 to 1 | 194.35 | 253.57 | 289.89 |
| 100 to 1 | 244.03 | 309.9 | 274.24 |
| LPS | 700.93 | 706.44 | 704.6 |
| | | | |
| Day 9 | 1 | 2 | 3 |
| None | 225.64 | 109.08 | 244.03 |
| 10 to 1 | 301.44 | 326.77 | 295.77 |
| 100 to 1 | 152.74 | 145.87 | 156.09 |
| LPS | 752.17 | 803.71 | 771.52 |
| | | | |
| Day 14 | 1 | 2 | 3 |
| None | 329.8 | 406.49 | 229.25 |
| 10 to 1 | 81.23 | 192.3 | 83.43 |
| 100 to 1 | 143.14 | 120.81 | 104.2 |
| LPS | 164.02 | 192.3 | 154.51 |

| | IL-10 [pg/mL] |
|---|---|
| Day 4 | 1 |
| None | 6.06 |
| 10 to 1 | 4.08 |
| 100 to 1 | 6.33 |
| LPS | 514.45 |
| | |
| Day 7 | 1 |
| None | 1.29 |
| 10 to 1 | 0.8 |
| 100 to 1 | 1.72 |
| LPS | 157.51 |
| | |
| Day 9 | 1 |
| None | 1.29 |
| 10 to 1 | 2.46 |
| 100 to 1 | 1.29 |
| LPS | 105.56 |
| | |
| Day 14 | 1 |
| None | 1.29 |
| 10 to 1 | 40 |
| 100 to 1 | 2.81 |
| LPS | 15.57 |

| 2 | 3 |
|---|---|
| 5.24 | 6.06 |
| 3.46 | 3.46 |
| 10.14 | 9.16 |
| 529.28 | 1204.69 |

| 2 | 3 |
|---|---|
| 0.8 | 0.8 |
| 0.8 | 1.29 |
| 1.29 | 1.72 |
| 153.01 | 151.62 |

| 2 | 3 |
|---|---|
| 1.72 | 2.1 |
| 2.1 | 2.29 |
| 2.46 | 1.72 |
| 97.2 | 87.74 |

| 2 | 3 |
|---|---|
| 1.29 | 2.98 |
| 43.18 | 35 |
| 1.91 | 2.29 |
| 21.82 | 15.8 |
