# Supplementary material for: Wear Particles Derived from Metal Hip Implants Induce the Generation of Multinucleated Giant Cells in a 3-Dimensional Peripheral Tissue-Equivalent Model
Source: PLoS One. 2015 Apr 20;10(4):e0124389. doi: 10.1371/journal.pone.0124389 (PMC4403993; doi:10.1371/journal.pone.0124389)
Supplement: S5 Fig — Particles were added at the time of gel polymerization at the ratio of 10:1 particles to PBMCs. Endothelial cells (EA) were grown on the gel to form a monolayer. Peripheral blood mononuclear cells (PBMCs) were seeded either on top of the monolayer. Cells were harvested by digesting the gel with RNA extraction buffer and proceed for RT-PCR as described in method. Data set is provided from two independent experiments. (PDF) [file pone.0124389.s005.pdf]

TRAP expression (number of copies)

Days	0	1	3	5	14
Exp-1	1	23	8	28	140
Exp-2	1	27	5	35	127

DC-STAMP expression (number of copies)

Days	0	1	3	5	14
Exp-1	1	56	5	13	25
Exp-2	1	85	6	26	55

GMCSF expression (number of copies)

Days	0	1	3	5	14
Exp-1	1	30	34	37	4
Exp-2	1	27	62	37	5
