# Supplementary material for: Wear Particles Derived from Metal Hip Implants Induce the Generation of Multinucleated Giant Cells in a 3-Dimensional Peripheral Tissue-Equivalent Model
Source: PLoS One. 2015 Apr 20;10(4):e0124389. doi: 10.1371/journal.pone.0124389 (PMC4403993; doi:10.1371/journal.pone.0124389)
Supplement: S6 Fig — As described above, co-cultures were set up at 10:1 ratio and incubated for 14 days. Cells were harvested by collagenase treatment, washed and intracellular stained with propidium iodide, FITC conjugated- TRAP and APC conjugated-DC-STAMP. The gating strategy is shown in this figure. (PDF) [file pone.0124389.s006.pdf]

FACSDiva Version 6.1.3

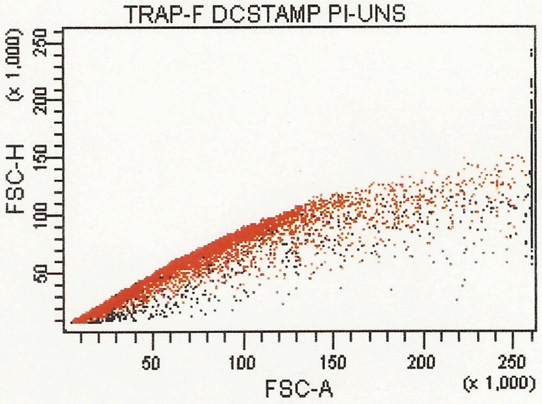

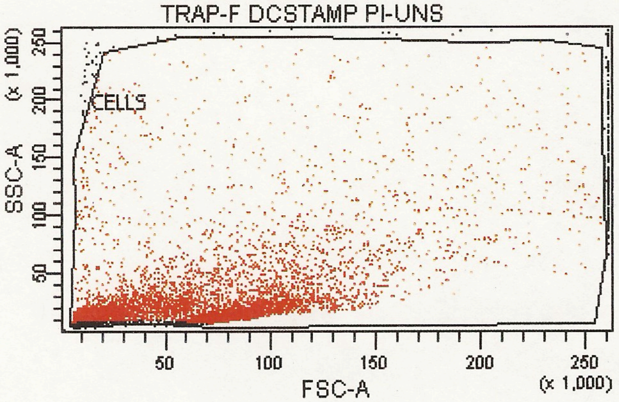

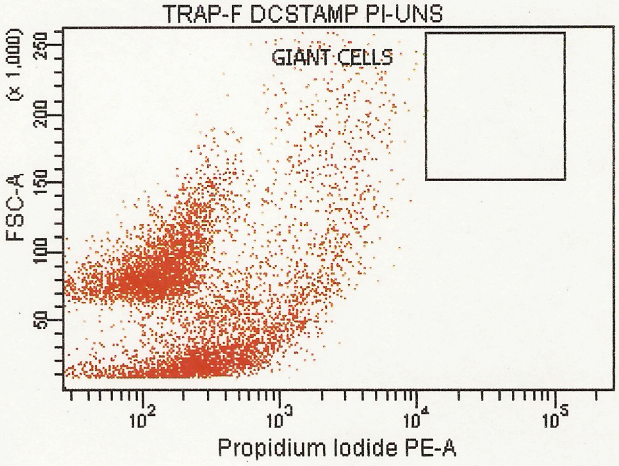

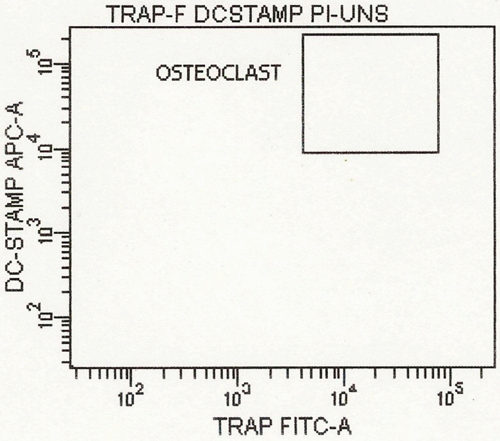

Tube: UNS

Population	#Events	%Parent	%Total
All Events	10,000	####	100.0
CELLS	7,105	71.0	71.0
GIANT CELLS	3	0.0	0.0
OSTEOCLAST	0	0.0	0.0

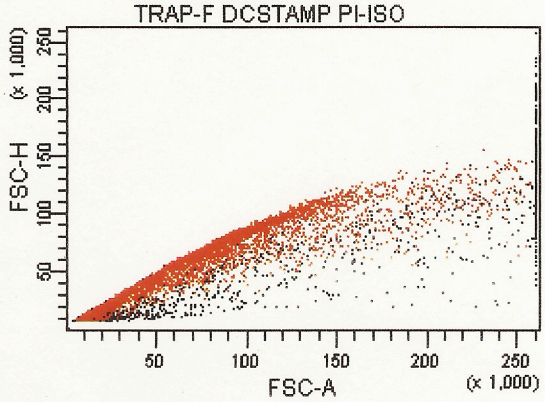

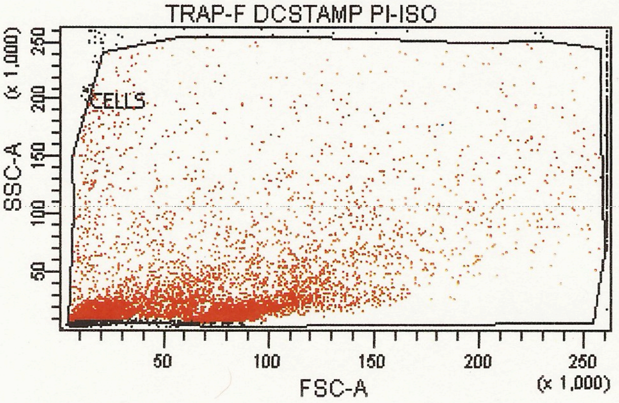

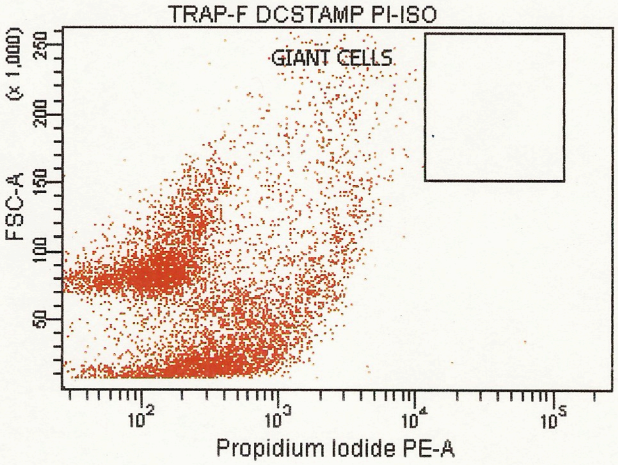

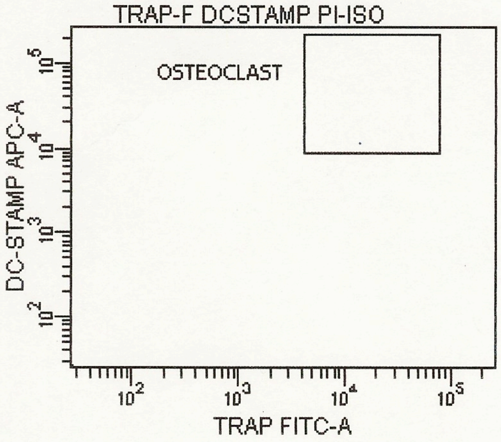

Tube: ISO

Population	#Events	%Parent	%Total
All Events	10,000	####	100.0
CELLS	7,167	71.7	71.7
GIANT CELLS	1	0.0	0.0
OSTEOCLAST	1	100.0	0.0

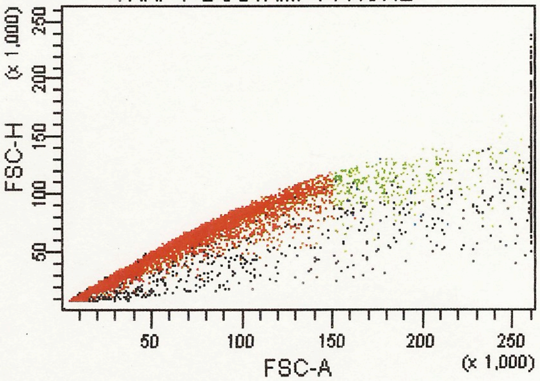

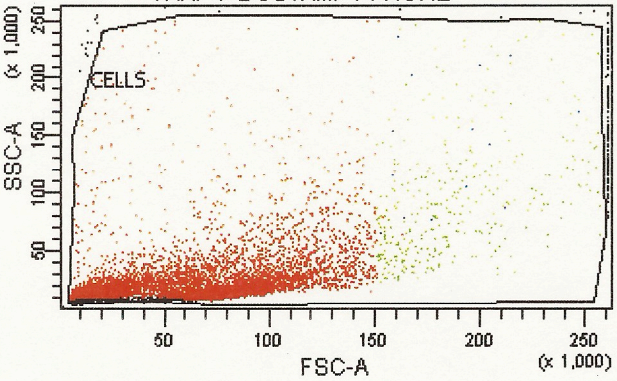

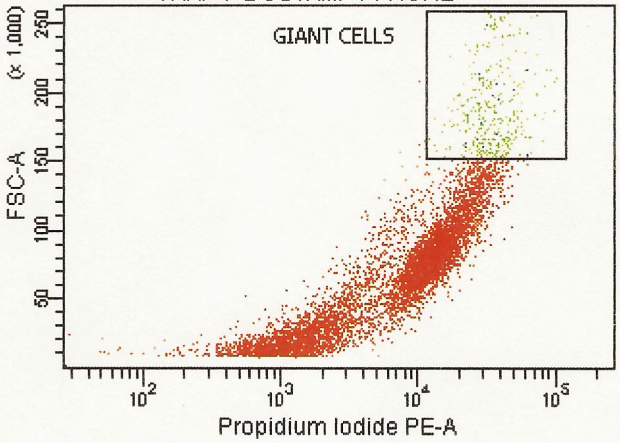

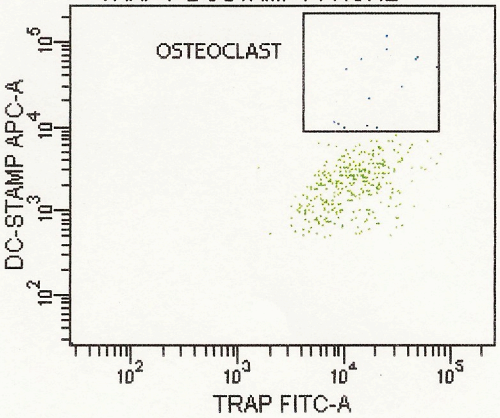

Tube: NONE

Population	#Events	%Parent	%Total
All Events	10,000	####	100.0
CELLS	6,842	68.4	68.4
GIANT CELLS	339	5.0	3.4
OSTEOCLAST	14	4.1	0.1

FACSDiva Version 6.1.3

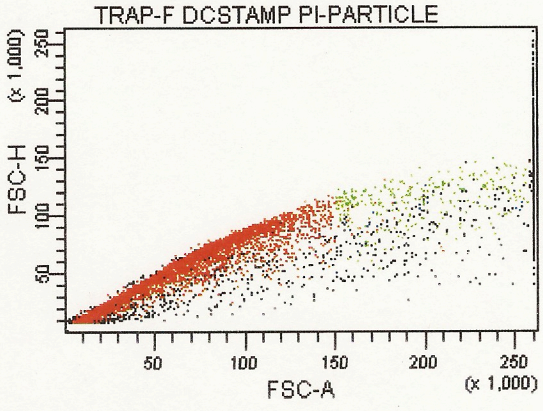

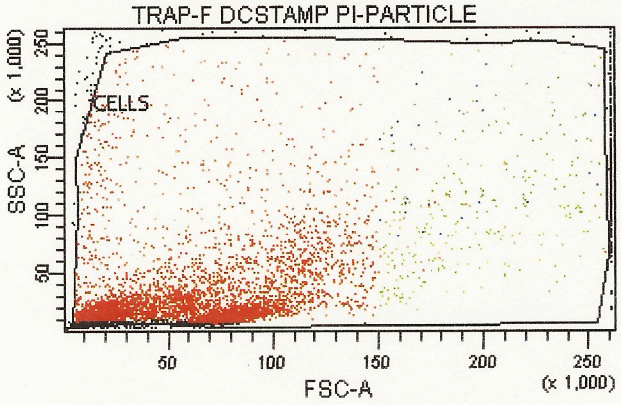

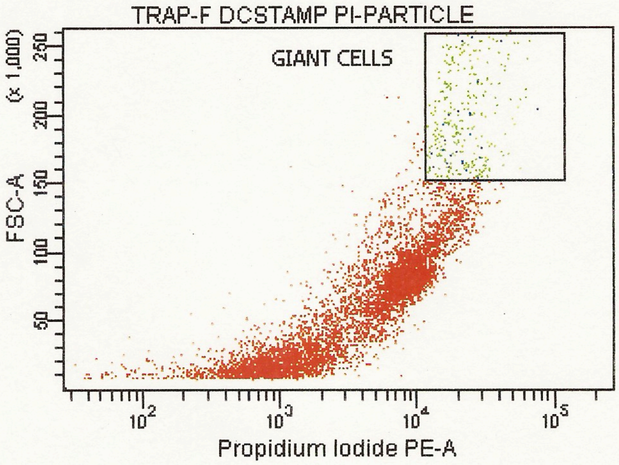

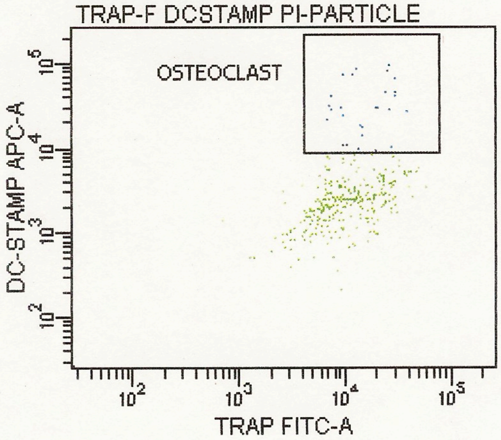

Tube: PARTICLE

Population	#Events	%Parent	%Total
All Events	10,000	####	100.0
CELLS	6,590	65.9	65.9
GIANT CELLS	299	4.5	3.0
OSTEOCLAST	27	9.0	0.3
