# Supplementary material for: Wear Particles Derived from Metal Hip Implants Induce the Generation of Multinucleated Giant Cells in a 3-Dimensional Peripheral Tissue-Equivalent Model
Source: PLoS One. 2015 Apr 20;10(4):e0124389. doi: 10.1371/journal.pone.0124389 (PMC4403993; doi:10.1371/journal.pone.0124389)
Supplement: S7 Fig — Particles were added at the time of gel polymerization at a ratio of 500:1, 100:1, or 10:1 or without particles (0:1) to PBMCs, as described previously, and incubated for 48h. Similar particle treated co-culture was set up in the absence of collagen gel in conventional 24 well plate. Cells were harvested with collagenase treatment from collagen gel, and stained with Live/Dead dye before acquisition on a flow cytometer. (PDF) [file pone.0124389.s007.pdf]

Batch Analysis Report

Run Date: 2/5/15 8:15 PM
Experiment: Debargh_apoptosis_2/5/15
User ID: SteveWood
Statistics Output: N/A
Worksheet PDF Output: D:\BDExport\FCS\22012015\Z-1133 Marburg peptides\Batch_Analysis_05022015201538.pdf

Specimen_001

Tube	Status	Run Time
UNS	OK	2/5/15 8:15 PM
2D None	OK	2/5/15 8:16 PM
2D 10:1	OK	2/5/15 8:16 PM
2D 100:1	OK	2/5/15 8:16 PM
2D 500:1	OK	2/5/15 8:16 PM
3D None	OK	2/5/15 8:16 PM
3D 10:1	OK	2/5/15 8:17 PM
3D 100:1	OK	2/5/15 8:17 PM
3D 500:1	OK	2/5/15 8:17 PM

FACSDiva Version 6.1.3

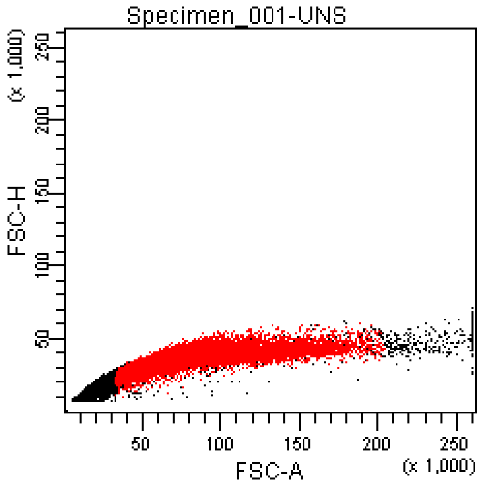

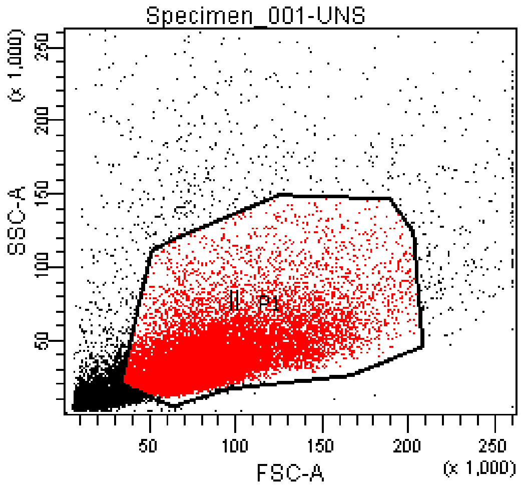

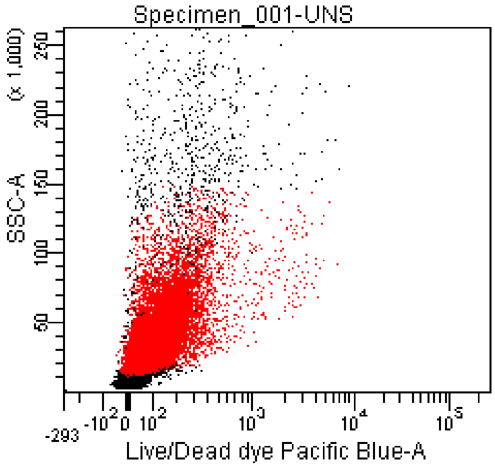

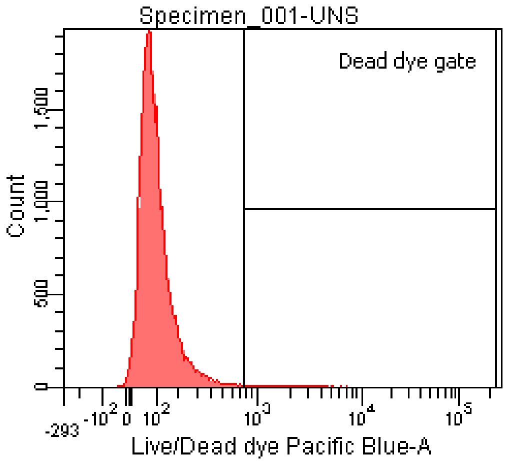

Tube: UNS

Population	#Events	%Parent	%Total
All Events	50,000	####	100.0
P1	31,074	62.1	62.1
Dead dye gate	159	0.5	0.3

FACSDiva Version 6.1.3

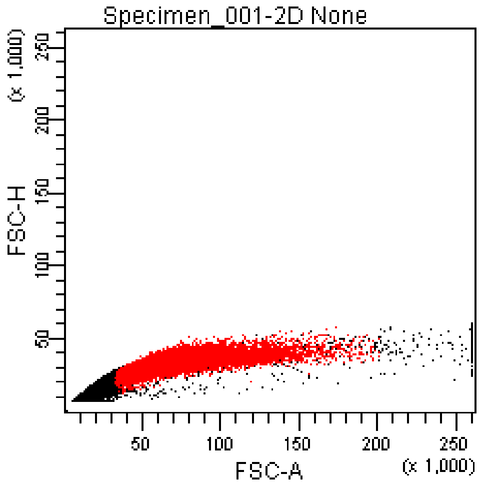

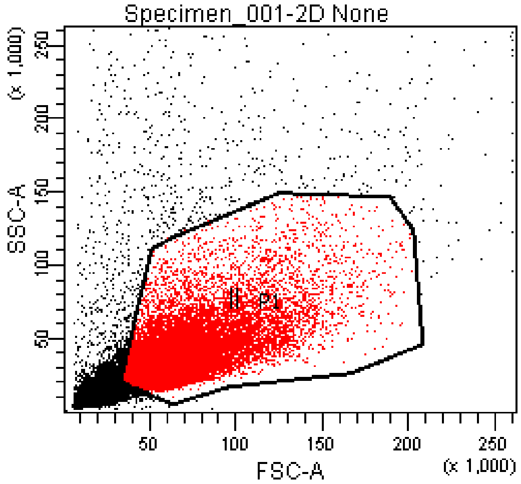

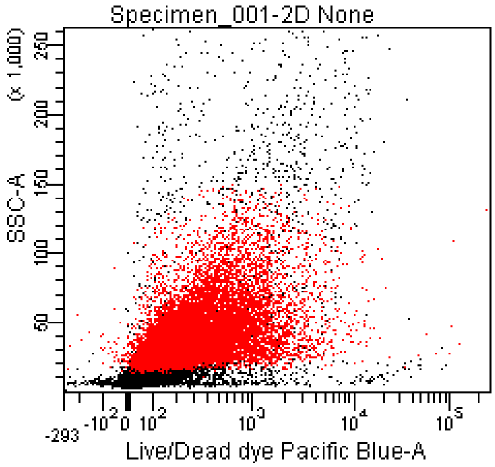

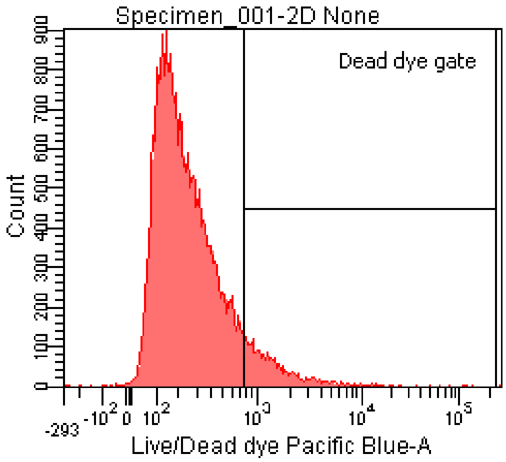

Tube: 2D None

Population	#Events	%Parent	%Total
All Events	50,000	####	100.0
P1	30,079	60.2	60.2
Dead dye gate	2,446	8.1	4.9

FACSDiva Version 6.1.3

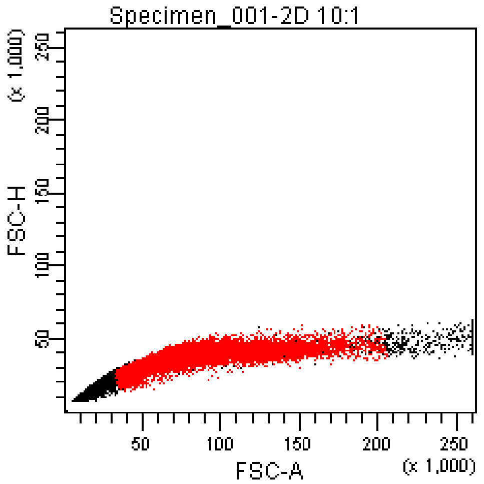

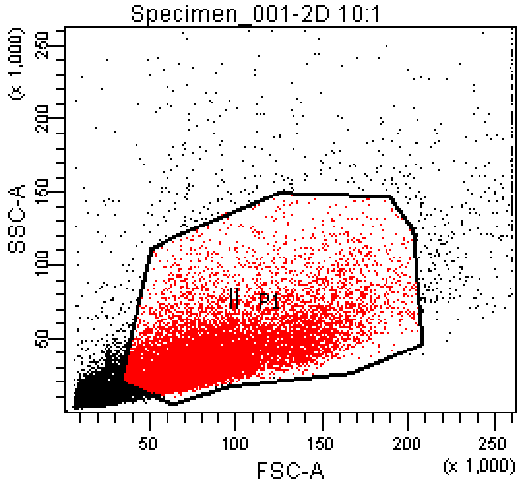

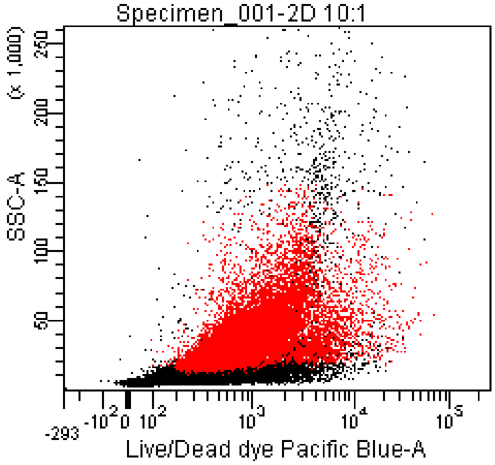

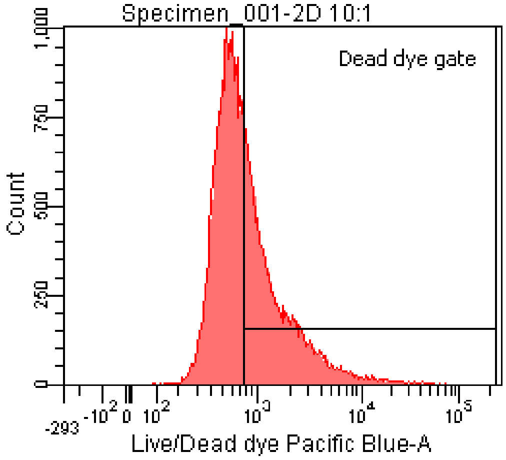

Tube: 2D 10:1

Population	#Events	%Parent	%Total
All Events	50,000	####	100.0
P1	31,628	63.3	63.3
Dead dye gate	12,738	40.3	25.5

FACSDiva Version 6.1.3

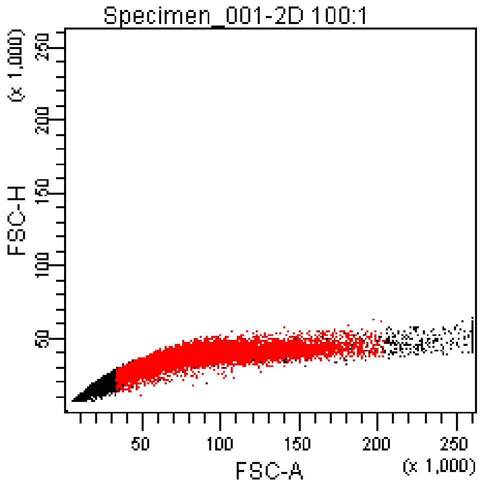

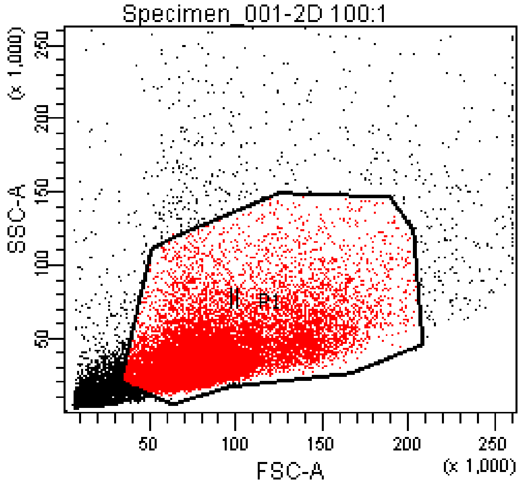

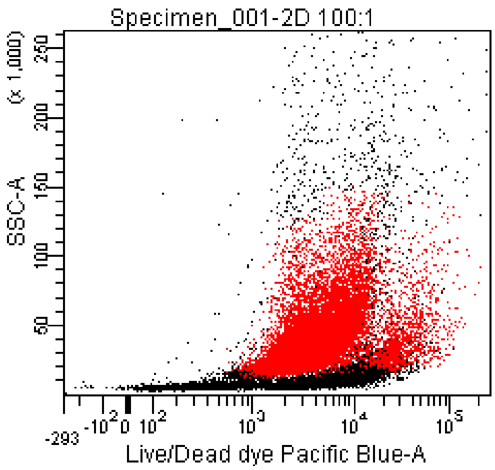

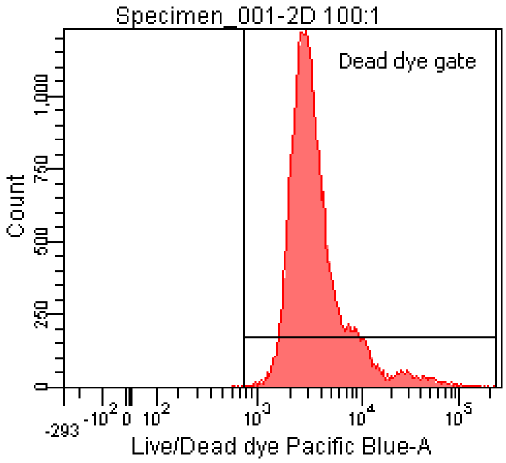

Tube: 2D 100:1

Population	#Events	%Parent	%Total
All Events	50,000	####	100.0
P1	32,015	64.0	64.0
Dead dye gate	32,008	100.0	64.0

FACSDiva Version 6.1.3

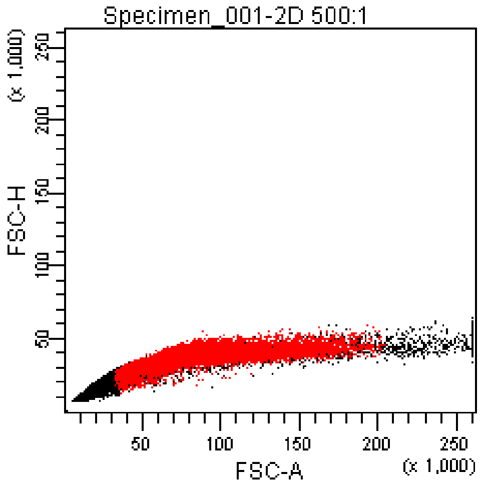

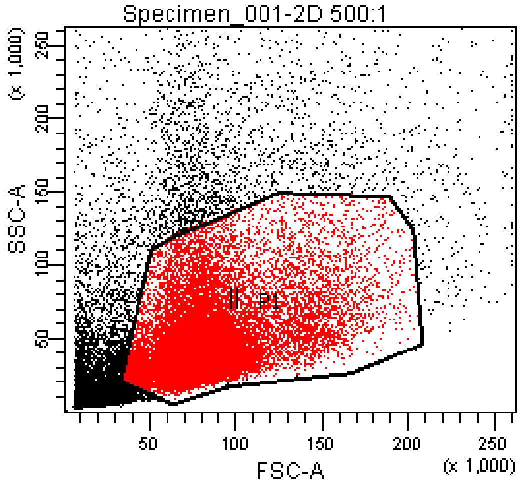

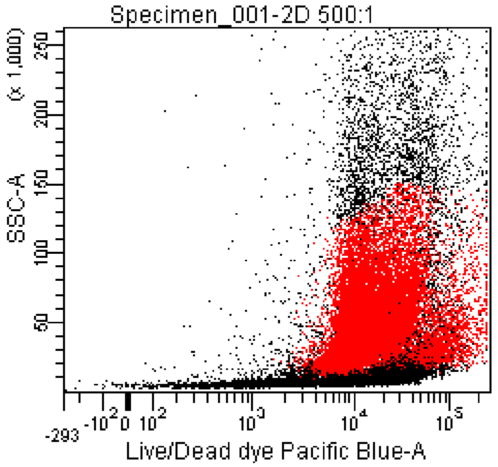

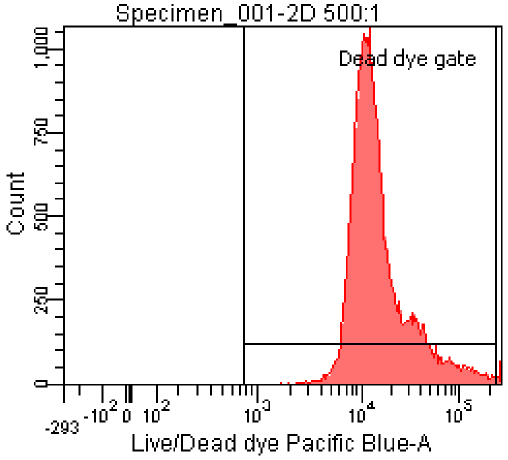

Tube: 2D 500:1

Population	#Events	%Parent	%Total
All Events	50,000	####	100.0
P1	26,791	53.6	53.6
Dead dye gate	26,696	99.6	53.4

FACSDiva Version 6.1.3

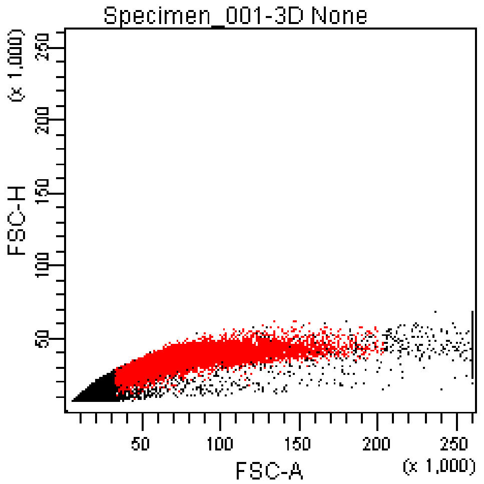

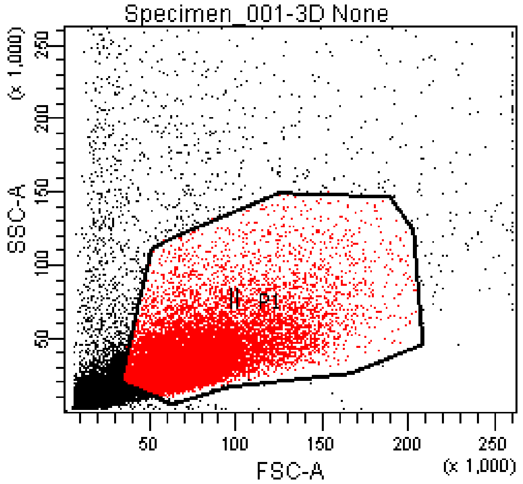

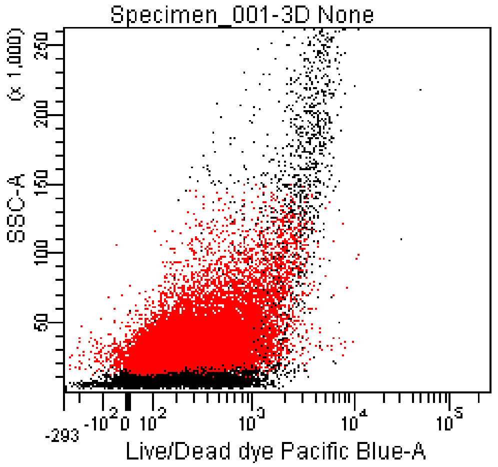

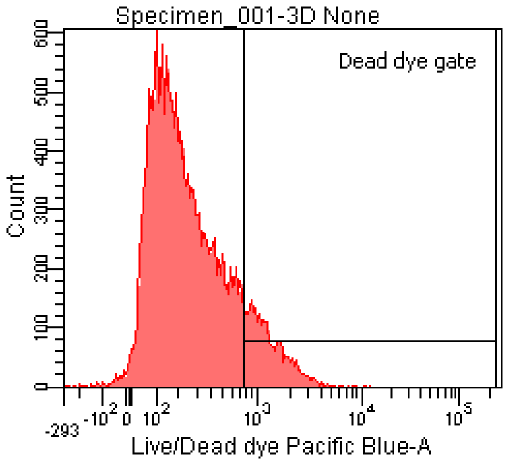

Tube: 3D None

Population	#Events	%Parent	%Total
All Events	50,000	####	100.0
P1	24,001	48.0	48.0
Dead dye gate	2,844	11.8	5.7

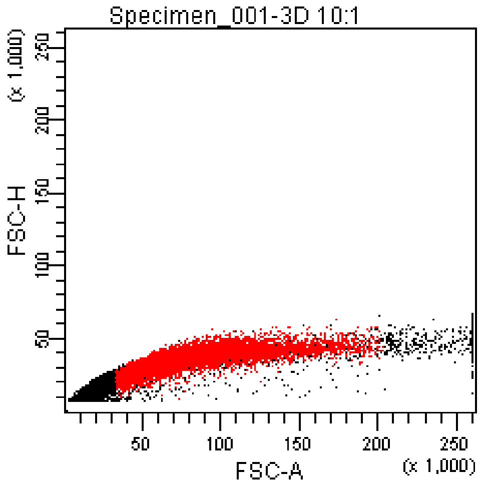

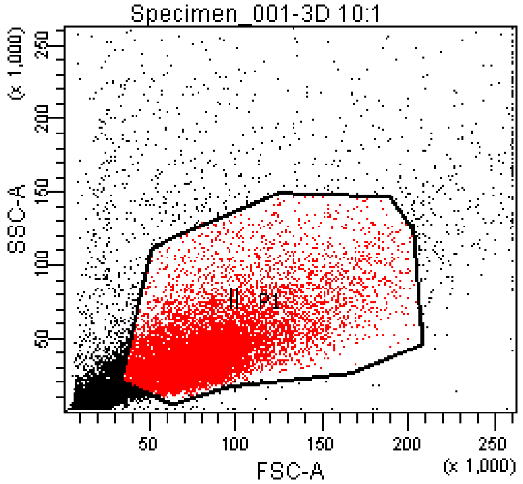

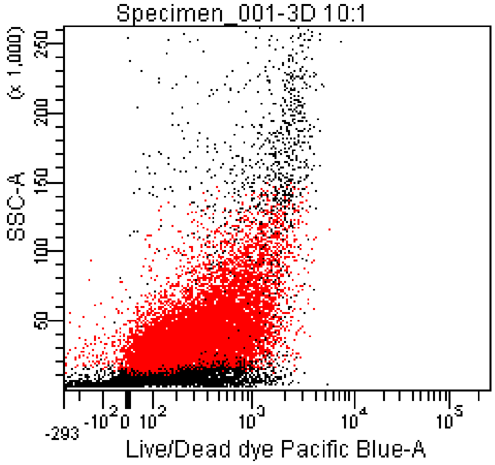

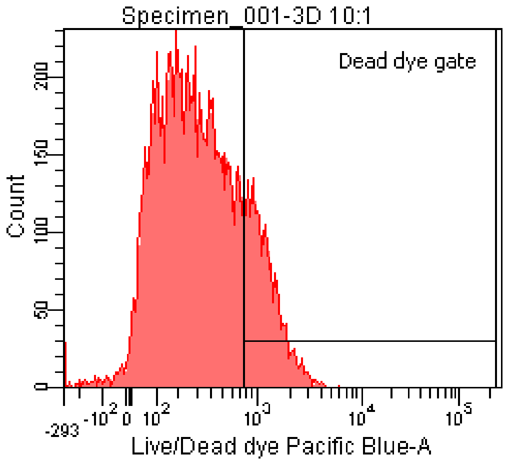

Tube: 3D 10:1

Population	#Events	%Parent	%Total
All Events	31,200	####	100.0
P1	13,164	42.2	42.2
Dead dye gate	2,311	17.6	7.4

FACSDiva Version 6.1.3

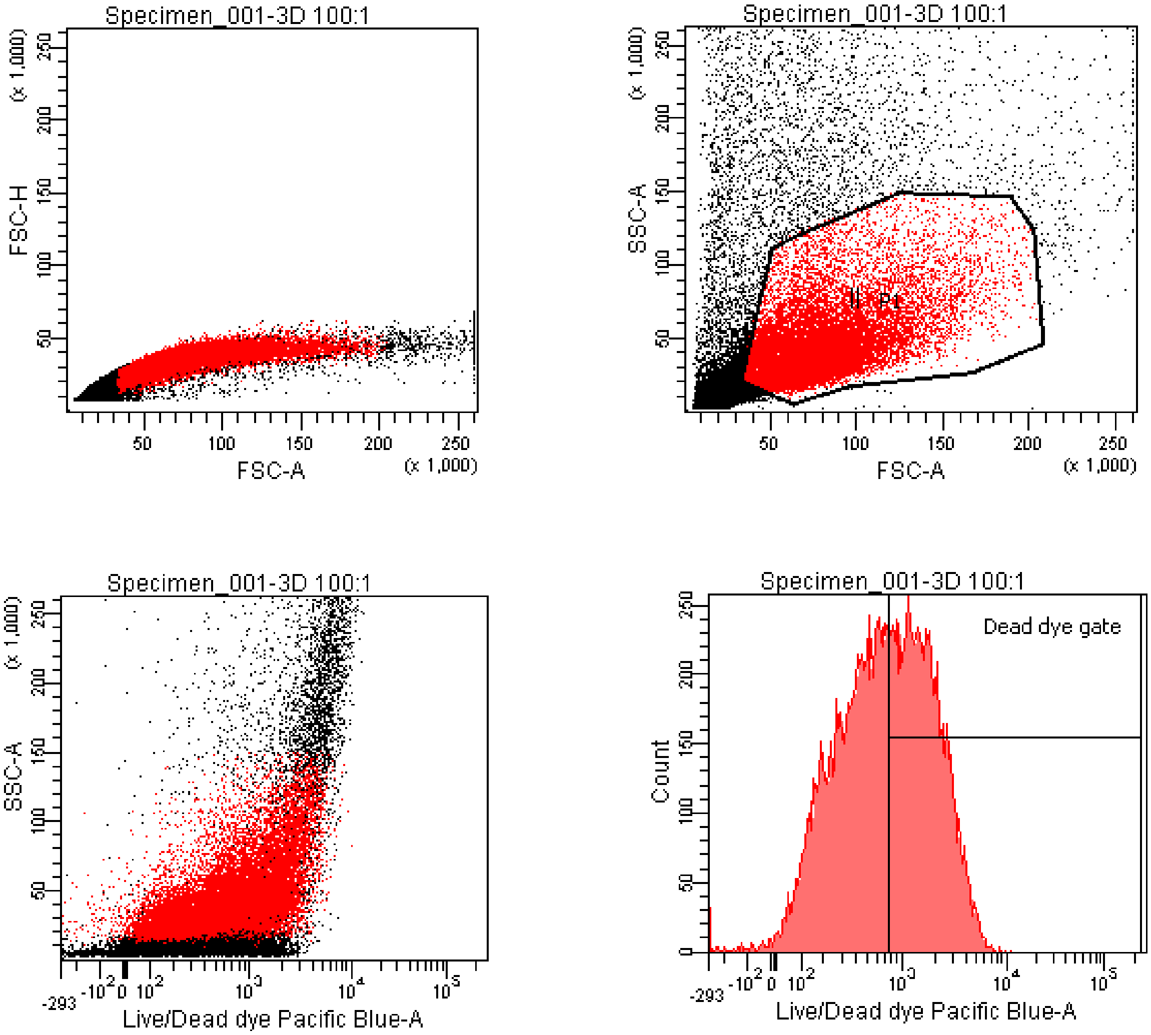

Tube: 3D 100:1

Population	#Events	%Parent	%Total
All Events	50,000	####	100.0
P1	17,887	35.8	35.8
Dead dye gate	8,433	47.1	16.9

FACSDiva Version 6.1.3

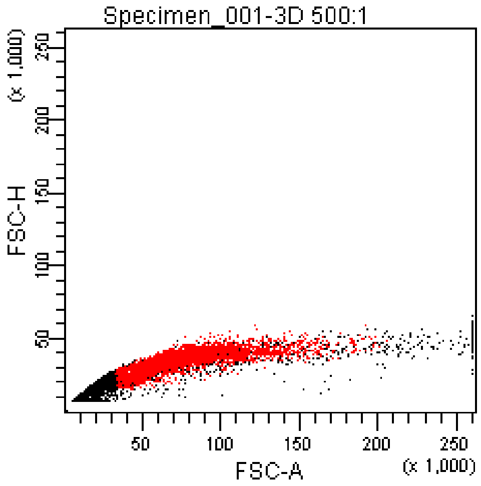

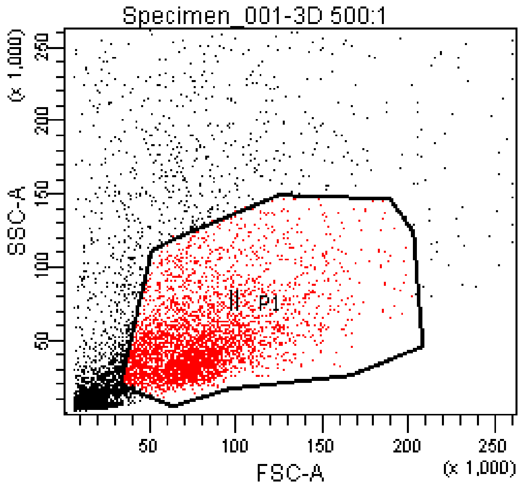

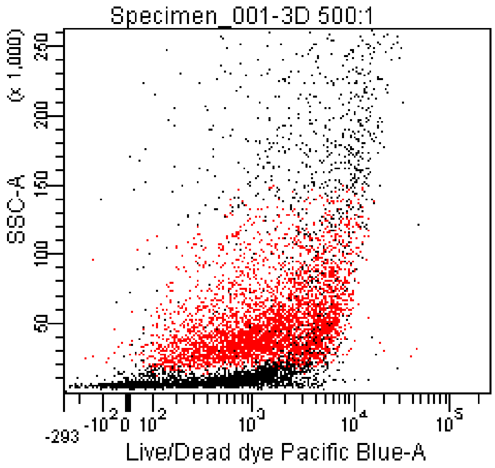

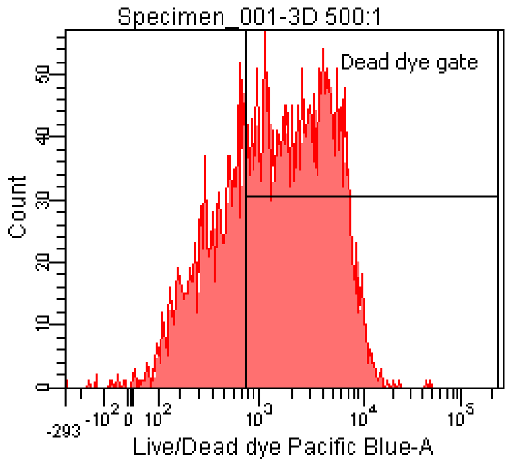

Tube: 3D 500:1

Population	#Events	%Parent	%Total
All Events	10,348	####	100.0
P1	3,930	38.0	38.0
Dead dye gate	2,691	68.5	26.0
